# Pre-Harvest Foliar Application of Chitooligosaccharide Modulates Aroma Quality of Cabernet Gernischt Wines

**DOI:** 10.3390/foods15122128

**Published:** 2026-06-12

**Authors:** Tengzhen Ma, Wenle Qiang, Lirong Zhang, Fei Yu, Baoquan Yuan, Yumei Jiang, Bo Zhang, Antonio Morata, Fumin Yang

**Affiliations:** 1 College of Food Science and Engineering, Gansu Agricultural University, Lanzhou 730070, China; 18742514819@163.com (W.Q.); zhanglirong@gsau.edu.cn (L.Z.); yufei@gsau.edu.cn (F.Y.); yuanbaoquan@gsau.edu.cn (B.Y.); jym316@gsau.edu.cn (Y.J.); zhangbo@gsau.edu.cn (B.Z.); 2 Chemistry and Food Technology Department, Universidad Polit’ecnica de Madrid, 28040 Madrid, Spain; antonio.morata@upm.es

**Keywords:** chitooligosaccharide, wine, volatile compounds, Cabernet Gernischt, aroma wheel

## Abstract

Pre-harvest foliar application of chitooligosaccharide (COS) was evaluated for its impact on the flavor quality of Cabernet Gernischt wines. COS was applied at the young berry (YB) and early veraison (EV) stages across the 2022 and 2023 vintages. Physicochemical parameters, phenolic compounds, color index, volatile composition, and sensory quality were systematically analyzed. In 2022, alcohol content and total acidity increased in both treatment groups, total phenols increased in the EV group but decreased in the YB group. In 2023, alcohol and acidity showed opposite trends between the two treatment groups, while phenolic compounds decreased. COS treatment increased wine lightness and yellow tone but reduced red tone and color saturation. In 2022 vintage, YB treatment increased total volatiles with 8.18% and terpenoids with 138.91%, while esters increasing by 34.72–53.60%. In 2023 vintage, total volatiles decreased by approximately 15%, with esters significantly decreasing by 26.60% (YB) and alcohols by 25.96% (EV), while fatty acids increased by 32.70% (EV). OPLS-DA identified key aroma compounds, including phenethyl acetate, ethyl caprate, heptyl acetate, and isoamyl acetate. Aroma wheel analysis showed that fruity and floral notes were enhanced in 2022 but reduced in 2023, with the EV stage consistently performing better. Overall, COS application at the early veraison stage represents a promising strategy for modulating wine flavor quality.

## 1. Introduction

Wine is a crucial alcoholic beverage worldwide, over the last several millennia, wine has played an important role in human culture [1]. Aroma is one of the most critical factors in wine quality evaluation. According to its origin, wine aroma can be categorized into varietal, fermentative, and aging aromas [2]. Among these, varietal aroma compounds derived from grape berries could contribute floral, fruity, and herbaceous notes, thus determine the stylistic and typical characteristics of the wine [3]. The biosynthesis and accumulation of aroma compounds and their precursors in grape berries are strongly influenced by grape cultivar, rootstock, climate conditions or ‘terroir’ (i.e., soil, climate, growing environment), and viticultural practices [4]. Since climate conditions are difficult to modify, and replanting new vine cultivars, clones, or rootstocks is time-consuming and costly, researchers and wine makers have shifted their attention toward identifying simple, economical, and controllable cultivation techniques to modulate the flavor quality of grapes and wines [5,6].

Recently, foliar application of plant growth regulators has drawn considerable attention as an effective strategy to improve grape quality [7,8]. Studies have shown that pre-harvest application of salicylic acid [9,10], methyl jasmonate [11,12], benzothiadiazole [13,14], abscisic acid [7,15], chitosan [8,16], 1-naphthaleneacetic [10], strigolactone [15] and Yeast extract [12,17] can affect the physicochemical parameters, phenolic compounds, and volatile composition of grape berries, thereby improving phenolic content, color index, aroma profiles and sensory quality of the resulting wines.

Chitooligosaccharide (COS), obtained through the deacetylation of chitosan under specific conditions, are widely used as biological inducers. Typically, COS consists of 2 to 10 units of N-acetylglucosamine or glucosamine linked by β-1,4-glycosidic bonds, and exhibits considerable safety, environmental friendliness, water solubility, affordability, and high biological activity [18]. This compound is not only cost-effective and eco-friendly but also capable of enhancing plant defense responses by activating multiple defense signaling pathways, thereby promoting secondary metabolic activities [19].

Foliar application of chitooligosaccharide could significantly increase the accumulation of total phenolic, total flavonoid, and total anthocyanin in the skins and seeds of Cabernet Franc grapes [18] and Cabernet Gernischt grapes [19]. Ma et al. reported that COS treatment regulates key genes in the shikimate pathway, thereby altering metabolic flux toward shikimate, tyrosine, and phenylalanine, which ultimately enhanced phenolic biosynthesis [19]. Singh et al. [20] also demonstrated that chitosan has a stimulatory effect on the accumulation of phenolic compounds, including anthocyanins, mediated by modifications in the transcription of key genes involved in their biosynthesis and transport in grape berries. Zhao et al. indicated that COS treatment improved the physicochemical indicators, phenolic substances, antioxidant capacity and color quality of grapes compared to control, thus enhancing the physical-chemical and sensory quality of the resulting wine [21]. Similarly, wines produced from Chitosan treated grapes significantly increased in the levels of total acetals and alcohols, and sensory analysis revealed higher overall acceptance [22].

Although the effects of chitooligosaccharide treatment on the chemical and volatile compositions of grape berries and resulting wines have been reported, research on the influence of pre-harvest COS application at different developmental stages on the flavor quality of the final wine has not yet been conducted. This study investigated the effects of pre-harvest foliar application of chitooligosaccharide on Cabernet Gernischt grapes at young berry and early veraison stages across two vintages, the physicochemical properties, phenolic profiles, volatile compounds, aroma profile and sensory quality of the elaborated wine were analyzed, thereby providing a theoretical basis for optimizing COS application protocols and improving Cabernet Gernischt wine quality.

## 2. Materials and Methods

### 2.1. Vineyard Site, Treatments, and Sampling

The experiment was conducted at the Grape Planting vineyard of Liangzhou Agricultural Ecological Technology Co., Ltd., in Liangzhou District, Wuwei City (102°24′ E, 36°46′ N), where Cabernet Gernischt grapes (*Vitis vinifera* L.) were planted in 1997. The vineyard is located at an altitude of 1500 m, with a temperate continental arid climate, characterized by more than 3000 annual sunshine hours, an average temperature of 8.8 °C, an annual effective accumulated temperature (>0 °C) of 3550 °C, a frost-free period of more than 150 days, and an average annual precipitation of 170.3 mm. The grapevines were planted in an east-west direction with a row spacing of 3.0 m and a within-row spacing of 1.0 m. Border vines were excluded from the experiment, and vines with generally uniform growth vigor were selected. Three biological replicates were established with per treatment and 20 grapevines per replicate at a random block layout design.

An electric sprayer was used to spray the entire canopy of the grapevines with a chitooligosaccharide (Hailongyuan Biotechnology Co., Ltd., Weifang, China) solution (0.1% *w*/*v*) containing 0.1% Tween 80 at the young berry stage (YB; E-L 32, 23 June 2022, and 1 July 2023) and the early veraison stage (EV; E-L 35, 15 August 2022, and 7 August 2023). The control group (CK) was sprayed with 0.1% (*v*/*v*) Tween-80 aqueous solution (Sigma-Aldrich, St. Louis, MO, USA). All sprays were applied until the leaf surface and fruit cluster surface were uniformly covered, and the solution began to drip from the vines. Grape berries were harvested on 24 September 2022, and 21 September 2023, respectively (E-L 38 stage). Subsequently, all grapes were transported to the laboratory on ice for wine making and analyzing.

### 2.2. Winemaking Process

50 kg of Cabernet Gernischt grapes in each groups were destemed and crushed, 100 mg/L of potassium metabisulfite and 20 mg/L of Color Plus pectinase (Enartis, San Martino Trecate, NO, Italy) were added, and the must was macerated at 8 °C for 24 h. Subsequently, a commercial *Saccharomyces cerevisiae* strain (Vintage Red, Enartis, San Martino Trecate, NO, Italy) was inoculated at a dose of 25 g/hL to initiate alcoholic fermentation, which was controlled at 20–25 °C. During fermentation, the tanks were pressed every 6 h until the residual sugar content dropped below 4 g/L, and 40 mg/L of potassium metabisulfite was added to stop the fermentation. The wine was transferred to a 30-L stainless steel tanks for sedimentation, and finally bottled and stored in a wine cellar (15 °C and 70% humidity) until analysis.

### 2.3. Physicochemical Analysis

The °Brix and total acidity content of the grapes, the alcohol content, total acidity, pH value, volatile acidity, and residual sugar of the wine were analyzed using a wine compositional analyzer (WineScan™, Foss, Hilleroed, Denmark).

Total phenols and total tannins were determined by Folin-Ciocalteu and Folin-Denis method respectively, while total anthocyanins were quantified by malvidin-3-glucoside equivalents using the pH differential method [18,23]. All measurements were performed in three replicates.

### 2.4. Color Parameter Analysis

Color parameters of the wine were assessed using the CIELab systems following the methods of Qiang et al. [18]. Wine sample was centrifuged at 12,000 r/min, and the supernatant was filtered through a 0.45 μm aqueous membrane and then transferred to a 2 mm quartz cuvette. A TU-1810 UV-Visible Spectrophotometer (Beijing Puxi General Instrument Co., Ltd., Beijing, China) was used to scan the visible light absorption spectrum of the wine sample from 400 to 780 nm at a scanning interval of 1 nm. The L*, a*, b*, C*ab, and H*ab were calculated by the absorbances at 450, 520, 570, and 630 nm.

### 2.5. Sensory Evaluation

The ethical approval for the sensory evaluation was obtained from the Ethics Committee of Gansu Agricultural University, and all panel members agreed to participate in the sensory study. The sensory evaluation was conducted in the Wine Tasting Room of Gansu Agricultural University by a professionally trained panel (4 males and 5 females), all participants had completed a comprehensive wine tasting course, which covered the theories and methods of wine evaluation. Additionally, the panel participated in a weekly training of Chinese wine evaluation system, which lasted for two hours each time, and passed the assessments. All the wine samples were randomly numbered and tested using a blind taste system at room temperature. The sensory characteristics of the wine samples were evaluated according to the Chinese wine evaluation system, an unstructured 10-point scale ranging from 1 (extremely low) to 10 (extremely high) was used. The attributes included appearance (clarity and color,10 points), aroma (intensity, elegance, and complexity, 30 points), taste (balance and structural, body and fullness, tannin texture and intensity, complexity, and aftertaste, 50 points), and overall profile (typicality, 10 points).

### 2.6. Volatile Compounds Analysis

#### 2.6.1. Volatile Compounds Extraction

For the 2022 wine samples, volatile compounds were extracted using a dispersive liquid-liquid microextraction (DLLME) method according to Liang et al. [24]. Briefly, 7 mL of wine, 10 μL of an internal standard solution (4-methyl-2-pentanol, 18.64 g/L in ethanol), 1200 μL of methanol (disperser solvent), and 600 μL of dichloromethane (extraction solvent) were added in a 15 mL glass centrifuge tube. The mixture was vortexed for 1 min. Subsequently, the organic phase was separated by centrifugation at 4000 rmp for 15 min, dried by hydrous sodium sulfate, and transferred to a 2 mL autosampler vial. Finally, 1 μL aliquot of the extraction solution was injected by an autosampler (Triplus RSH, Thermo Fisher Scientific, San Jose, CA, USA) with a solvent delay time of 7 min.

For the 2023 wine samples, volatile compounds were extracted by headspace solid-phase microextraction (HS-SPME) following the procedure described by Ma et al. [25] 5 mL of wine, 1.5 g sodium chloride, and 10 µL of 2-octanol (internal standard, with the concertation of 1000 mg/L) were transferred to a 15 mL headspace vial. The vial was equilibrated at 40 °C on a magnetic stirrer for 30 min, and the volatile components in headspace were adsorbed by a DVB/carboxen/PDMS fiber (50/30 µm, fiber length 1 cm, Supelco, Bellefonte, PA, USA) coupled with a manual holder. All extractions were performed in three independent replicates, with each replicate measured in duplicate (*n* = 6).

#### 2.6.2. Chromatographic and Mass Spectrometric Conditions

A gas chromatography-mass spectrometer system(TRACE 1310, ISQ, Thermo Fisher Scientific, San Jose, CA, USA) equipped with a DB-WAX column (60 m × 0.25 mm × 0.25 µm, Agilent Technology, Santa Clara, CA, USA) was used for volatile analysis. The SPME fiber or the extraction solution were introduced into the injector port of the GC–MS system for desorption and analysis at 250 °C for 7 min in splitless mode, helium (purity 99.9%) was used as the carrier gas at a constant flow rate of 1.0 mL/min. The GC oven temperature started at 40 °C for 5 min, then increased to 220 °C and held for 10 min. The electron ionization (EI) voltage was set to 70 eV, and the mass range were 50 to 350 *m*/*z*. The transfer line temperature was 230 °C and the ion source temperature was 250 °C.

#### 2.6.3. Qualitative and Quantitative Analysis

Detected volatile compounds (obtained as peaks and mass spectra) were identified by comparing their mass spectra with those in the National Institute for Standards and Technology library (NIST 14, search version 2.0). Retention index was calculated by C6-C24 n-alkane series (Supelco, Bellefonte, PA, USA) and compared with the NIST standard reference database. Volatile compounds with standards were quantified using calibration curves, other compounds without standards were calculated as µg/L of internal standard.

### 2.7. Aroma Wheel Analysis

The odor activity value (OAV), defined as the ratio of the concentration of a volatile compound above its perception threshold, was calculated as described by previous studies [25]. Aroma series including fruity, chemical, floral, caramelized, fatty, spicy, nutty, herbaceous, oxidized, earthy, woody, microbiological, and pungent scent descriptors were used to categorize the overall aroma profile of the Cabernet Gernischt wines by suming the OAV of volatiles with an OAV > 0.1 [26].

### 2.8. Statistical Analysis

Microsoft Excel 2021 was used for data recording. All statistical analyses were performed using SPSS version 19.0 (IBMSPSS Inc., Armonk, NY, USA). Data for physicochemical parameters, color index, volatile compounds and sensory scores were analyzed using analysis of variance (ANOVA). Significant differences were determined using Tukey’s range test at *p* < 0.05. In addition, orthogonal least squares-discriminant analysis (OPLS-DA) was performed using the Simca 14.1 program (Umetrics, Umeaa, Sweden).

## 3. Results

### 3.1. General Oenological Parameters of Wines

Preharvest chitooligosaccharide treatments significantly influenced the general oenological parameters of Cabernet Gernischt grapes and the resulting wine (Table 1). Compared to the control group, the weight of 100 berries showed an increase of 1.31–14.53% in 2022 and a decrease of 4.16–6.54% in 2023 vintage. The °Brix of both vintages increased by 3.97–22.77%, with YB treatment in 2023 vintage showed the largest increasing. The content of total acidity decreased by 4.17–13.50%, only in YB group observed significant influence.

The alcohol content of the wines ranged from 10.53% vol to 12.33% vol. Compared with the control wine, EV treatment significantly increased alcohol content in 2022 by 3.67%, while in 2023, YB treatment increased alcohol content by 6.38% and EV treatment decreased by 9.15%. Relative to the control, total acidity in YB and EV treatments significantly increased by 1.66% and 4.40% in 2022 vintage. However, in 2023 vintage, total acidity significantly decreased by 8.15% in YB wine and increased by 4.28% in EV wine. Contrary to the trend observed for total acidity, the pH value of the wines showed no significant difference in 2022 vintage, however, YB treatment significantly increased by 9.74% in 2023 vintage, while no significant difference was observed between the EV and CK groups. In addition, although there were statistically significant differences in residual sugar and volatile acid among wine samples, their contents (below 0.5 g/L and 0.58 g/L, respectively) were scarcely impact on wine quality.

In 2022 vintage, EV treatment showed the highest total phenolic content, which was significant increased by 11.94% and 31.19% compared to CK and YB treatments, respectively (Table 1). In contrast, no significant difference was observed between the CK and YB treatment group in 2023 vintage, however, the EV treatment exhibited a significant reduction of 17.75% compared to CK. Total tannin content also varied with vintage. Compared to the CK wine, the YB treatment in 2022 and EV treatment in 2023 significantly decreased total tannin content by 36.62% and 24.66%. Although no significant difference was observed in anthocyanin content among 2022 wine samples, YB and EV treatment in 2023 vintage showed significantly decreased in anthocyanin content by 17.65% and 35.25%, respectively.

### 3.2. Wine Color Assessment

Color is an important quality attribute for red wine and can be comprehensively evaluated through CIElab parameters. As shown in Table 2, the lightness values (L*) of wine samples from the chitooligosaccharide treatment groups significantly increased by 0.79–6.90% across different vintages, indicating an overall lighter wine color in the treated groups. The green-red axis (a*) and chromaticity (C*ab) showed similar trends. In 2022 vintage, the YB treatment significantly reduced by 28.02% and 27.99%, respectively, while the EV treatment showed no significant influence. In 2023 vintage, a* significant decreased by 21.24–42.53%, while C*ab showed a 21.13–42.51% decrease in the treatment groups. For the yellow-blue axis (b*), both treatments in 2022 resulted in significantly higher values than the control wine, whereas in 2023 vintage, only the YB treatment significantly increased. In addition, the hue (H*ab) of all wine samples was approximately 0° (range: 0.038–0.067), indicated a red tone (Table 2).

Additionally, correlation analysis revealed that L* value was significantly negatively correlated with a* value, Cab value, and the contents of anthocyanins, total phenols, and tannins. The a* value was significantly positively correlated with Cab value as well as with anthocyanin and total phenol contents. Furthermore, b* value showed a significant positive correlation with Hab value, while Cab value was significantly positively correlated with total phenol and tannin contents (Figure S1). Overall, chitooligosaccharide treatment enhanced lightness and yellow hue while reducing red hue and chromaticity, but the effects varied with treatment stage and vintage.

### 3.3. Influence on Sensory Attributes of Wine

**Figure 1:**
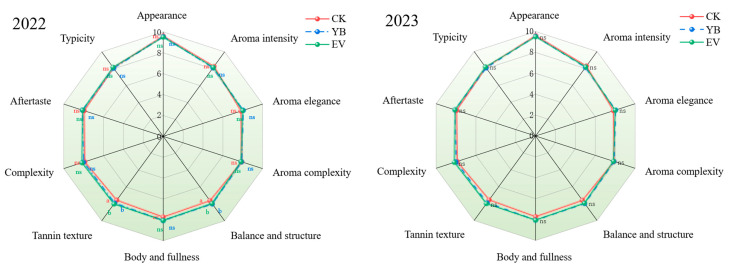
Impact of chitooligosaccharide treatment on sensory attributes of Cabernet Gernischt wines. Different letters within columns in each vintage indicate significant differences between the treatments, ns means no significant difference were observed (Tukey’s range test, *p* < 0.05).

Sensory evaluation showed that all wine samples presented clear appearance with a purple-red color, rich and elegant aroma, harmonious body, fine tannins and a long aftertaste, exhibiting the typical flavor characteristics of Cabernet Gernischt wine (Figure 1). In 2022 vintage, the CK wine obtained significantly lower scores for taste in terms of balance and structure, and tannin texture compared to YB and EV wines. For both vintages, no significant differences were observed among all groups in appearance, aroma intensity, aroma elegance, aroma complexity, body and fullness, complexity, aftertaste and typicity. In summary, pre-harvest chitooligosaccharide treatment did not exert significant influence on the overall sensory quality of young Cabernet Gernischt wine, and only slightly improved taste coordination and tannin quality.

In the sensory evaluation, the differences between the treatment group and the control group of the new wines are relatively small, especially in the same vintage, the same vineyard, the same cultivation management, as well as the same brewing techniques, which makes it difficult for the panels to fully reflect the subtle effects brought from the oligosaccharide treatment. Besides, the current method of aroma assessment only focused on the aroma intensity, elegance and complexity, lack in quantitative descriptors analysis. Further evaluation of sensory attributes during aging, and an in-depth quantitative descriptors analysis is required to systematically assess the long-term regulatory effect of chitooligosaccharide treatment on wine sensory characteristics.

### 3.4. Impacts on Volatile Composition of Cabernet Gernischt Wines in 2022 Vintage

#### 3.4.1. Influence on Categories and Contents

**Figure 2:**
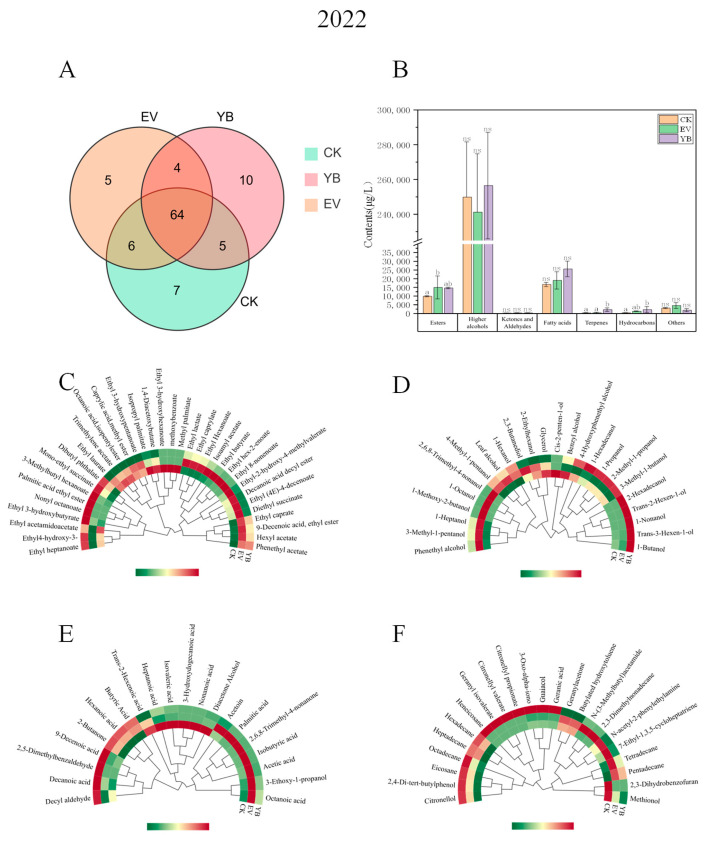
Volatile composition of Cabernet Gernischt wine in 2022 vintage. (**A**). Venn diagrams, (**B**). Contents of volatile compounds, (**C**). Heat maps of esters, (**D**). Heat maps of higher alcohols, (**E**). Heat maps of aldehydes, ketones and fatty acids, (**F**). Heat maps of terpenes and other compounds. Different letters within columns in each vintage indicate significant differences between the treatments, ns means no significant difference were observed (Tukey’s range test, *p* < 0.05).

Volatile composition is one of the most important indicators for evaluating wine aroma quality. In 2022 vintage, dispersive liquid-liquid microextraction (DLLME) coupled with gas chromatography-mass spectrometry (GC-MS) were used to determine the volatile compounds of the wine samples. A total of 101 volatile compounds were identified, with 83, 82 and 79 volatile compounds identified in the YB, CK and EV wine samples, respectively (Figure 2A). Among these, 64 aroma compounds were detected in all three wine groups, including 19 esters, 15 higher alcohols, 5 aldehydes and ketones, 10 fatty acids, 4 terpenes and C_13_-norisoprenoids, 6 hydrocarbons, and 6 other compounds. The numbers of uniquely detected compounds were 10, 7, and 5 in the YB, CK, and EV wines, respectively (Table S1).

Compared with the CK wine, total volatile content of the YB wine significantly increased by 8.18%, whereas no significant difference was observed for the EV group. Compared to the control wine, YB and EV wine showed significant increase in total content of esters by 34.72% and 53.60%, total aldehydes and ketones by 17.80% and 21.55%, total hydrocarbons by 371.48% and 165.28%, and total terpenes and C_13_-norisoprenoids by 138.91% in YB group. In contrast, the total contents of higher alcohols, fatty acids and other compounds showed no significant differences among wine samples (Figure 2B).

#### 3.4.2. Influence on Esters

Esters represented with the highest species diversity and content of the volatiles in the presented wine samples. In 2022 vintage, 28, 25 and 27 esters were detected in the CK, YB and EV wines, respectively. Ethyl 3-hydroxyhexanoate, nonyl octanoate, methyl palmitate and ethyl palmitate were only detected in CK wine, ethyl hex-2-enoate and mono-ethyl succinate were exclusive to EV wine, and decanoic acid decyl ester was only found in YB wines (Figure 2C).

Isoamyl acetate (3542.50 μg/L), ethyl hexanoate (1388.38 μg/L) and ethyl octanoate (1318.10 μg/L) constituted the dominant ester profile by average concentration (Table S1). Compared to the control wine, phenethyl acetate significantly increased by 71.16% and 75.12% in YB and EV groups, respectively. The concentration of isoamyl acetate, ethyl hexanoate, ethyl lactate, ethyl octanoate, ethyl decanoate and trans-4-decenoate showed significant increased of 13.60–88.32% in EV treatment, and these compounds also showed higher content of 5.48–44.31% in YB group, though the differences were not significant (Figure 2C).

#### 3.4.3. Influence on Higher Alcohols

In 2022 vintage, a total of 20, 20 and 18 alcohols were detected in CK, YB and EV wines, respectively. cis-2-Pentenol and 1-methoxy-2-butanol were only detected in CK and EV wine respectively, while trans-2-hexen-1-ol, 2-hexadecanol and 1-nonanol were only found in YB wine (Table S1). Isoamyl alcohol was the most abundant alcohol component in Cabernet Gernischt wine, followed by phenethyl alcohol, isobutanol, hexanol and 2,3-butanediol. Notably, 3-hexen-1-ol increased significantly by 15.65% and 25.11% in the YB and EV groups, while 3-Methyl-1-pentanol and 3-hexen-1-ol significantly elevated by 12.82% in EV group and 17.09% in YB group, respectively. Other alcohols, such as propanol, isobutanol and isoamyl alcohol, showed no significant differences among three wines (Figure 2D).

#### 3.4.4. Influence on Aldehydes, Ketones and Fatty Acids

7 aldehyde and ketone compounds were detected in 2022 vintages, with 2,5-dimethylbenzaldehyde was only identified in YB wine. Additionally, compared to the CK and YB wine, the contents of 1,3-propanediol monoethyl ether and 3-hydroxy-2-butanone in EV wine were significantly increased by 22.62–48.20% and 18.84–68.41%, respectively (Figure 2E).

A total of 13 fatty acids were detected, among which, 3-hydroxylauric acid and nonanoic acid were only identified in the CK wine, while hexadecanoic acid was only detected in the EV wine. Octanoic acid, hexanoic acid and decanoic acid were the predominant fatty acids. The content of butyric acid in the YB wine significantly increased by 41.21% compared to the CK wine, but no significant difference was observed compared to the EV wine. Acetic acid, isobutyric acid, isovaleric acid, hexanoic acid, octanoic acid, decanoic acid and 9-decenoic acid showed no significant differences among treated wines (Figure 2E).

#### 3.4.5. Influence on Terpenes, C_13_-Norisoprenoids, Hydrocarbons and Other Compounds

A total of seven terpene and C_13_ norisoprenoid compounds were detected in 2022 wine samples, among which geranyl isovalerate, citronellyl propionate and citronellyl valerate were only detected in YB wine. Citronellol, geranyl acetone, 9-Hydroxy-4,7-megastigmadien-3-one, and geranic acid exhibited no significant differences among different wine samples (Figure 2F).

A total of nine hydrocarbons and seven other compounds were detected in the 2022 wine samples. Among them, heneicosane was only found in the YB wine, 2,3-dimethylnonadiene was only detected in the EV wine, while eicosane and 2,3-dihydrobenzofuran were not detected in the CK wine. The contents of pentadecane and hexadecane in the YB group significantly increased by 66.98% and 447.19%, while in EV wine, 7-ethyl-1,3,5-cycloheptatriene, pentadecane, hexadecane and N-isopentylacetamide significantly increased by 46.22–338.01% (Figure 2F).

### 3.5. Impacts on Volatile Composition of Cabernet Gernischt Wines in 2023 Vintage

#### 3.5.1. Influence on Categories and Contents

**Figure 3:**
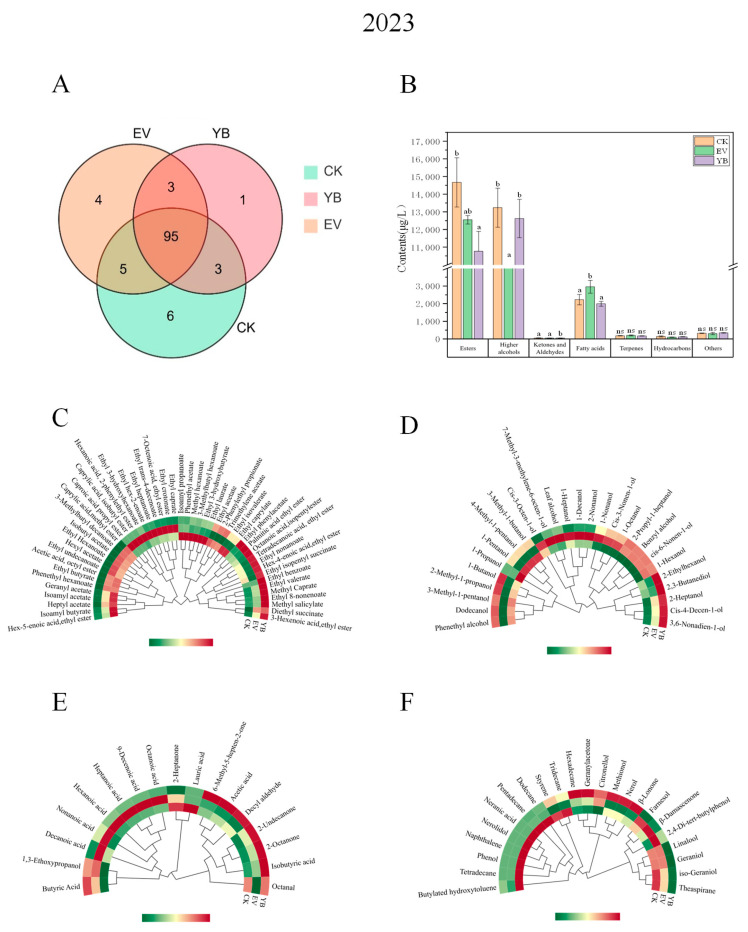
Volatile composition of Cabernet Gernischt wine in 2023 vintage. (**A**). Venn diagrams, (**B**). Contents of volatile compounds, (**C**). Heat maps of esters, (**D**). Heat maps of higher alcohols, (**E**). Heat maps of aldehydes, ketones and fatty acids, (**F**). Heat maps of terpenes and other compounds. Different letters within columns in each vintage indicate significant differences between the treatments, ns means no significant difference were observed (Tukey’s range test, *p* < 0.05).

In 2023 vintage, headspace solid-phase microextraction (HS-SPME) coupled with gas chromatography-mass spectrometry (GC-MS) were used to determine the volatile compounds of the wine samples. A total of 116 volatile compounds were detected, with 109, 107 and 102 volatile compounds identified in the CK, EV and YB wines, respectively (Figure 3A). Among these, 95 aroma components were found in all three wines, including 39 esters, 23 higher alcohols, 6 aldehydes and ketones, 9 fatty acids, 8 terpenes and C_13_-norisoprenoids, 7 hydrocarbons, and 3 other compounds. The numbers of uniquely detected compounds were 6, 4, and 1 in the CK, EV and YB wines, respectively (Table S2). The total volatile contents of the YB and EV treatments showed significantly decreased of 15.36% and 15.76% compared to the CK group.

Compared to the control wine, YB and EV wines showed significant decrease in total esters content by 26.60% and 14.44%, respectively. Notably, in the EV wine, total alcohols decreased by 25.96% while total fatty acids increased by 32.70%. In addition, total aldehydes and ketones significantly increased by 22.17% in YB group. No significant difference was observed in total terpenes, total hydrocarbons, and other compounds (Figure 3B). The results indicated that vintage and application stage significantly affected the numbers, total content, and unique components of wine aroma substances. Therefore, further analysis of their variation characteristics by functional group is necessary.

#### 3.5.2. Influence on Esters

In 2023 vintage, 44, 43 and 46 esters were detected in the CK, YB and EV wines, respectively. Ethyl benzoate only detected in YB wine, while propyl hexanoate, isobutyl octanoate and 2-phenethyl hexanoate were specific to EV wine. In addition, ethyl valerate was not detected in CK wine, geranyl acetate and 2-phenethyl hexanoate were not identified in YB group, 1,3-propanediol diacetate and 2-phenethyl propionate were not identified EV group (Figure 3C).

Ethyl octanoate (3404.74 μg/L) showed the highest content, followed by isoamyl acetate (3172.21 μg/L), ethyl hexanoate (3172.21 μg/L) and ethyl acetate (2248.37 μg/L), these esters constituted the dominant profile by average concentration (Table S2). Compared to control wine, COS treatments significantly reduced the contents of phenethyl acetate and heptyl acetate, with decreases of 32.18% and 61.91% in YB groups, and 24.97% and 17.63% in EV groups. In contrast, ethyl 8-nonenoate increased significantly by 37.53% in YB and 17.13% in EV group. In the YB wine, isobutyl acetate, ethyl butyrate, ethyl butyrate, hexyl acetate, heptyl acetate, caprylic acid methyl ester and ethyl caprylate decreased significantly by 13.18–65.07%, while methyl decanoate and methyl salicylate increased by 24.21% and 26.30%, respectively. In the EV wine, ethyl heptanoate, ethyl hex-2-enoate and hexyl acetate increased significantly by 7.32–19.08%, whereas ethyl acetate and ethyl isovalerate decreased by 32.03% and 42.18%. Compared to the YB wine, EV wine presented significantly lower contents of ethyl nonanoate and ethyl palmitate by 10.90% and 76.21%, but increased 22.16% in ethyl crotonate. Other 22 esters showed no significant differences among wines (Figure 3C).

#### 3.5.3. Influence on Higher Alcohols

In 2023 vintage, 25 alcohols were identified, of which 23 were detected in all three wines. 1-Propanol and 7-methyl-3-methylene-6-octen-1-ol were only detected in CK and EV wine, respectively. The most abundant alcohols were isoamyl alcohol, followed by phenethyl alcohol, isobutanol, 1-hexanol, 1-heptanol, octanol and nonanol. The contents of hexanol, octanol, cis-6-nonen-1-ol, 2-propyl-1-heptanol and benzyl alcohol showed significant increase in chitooligosaccharide treated wine, ranging from 22.54–81.87% in YB group and 23.25–100.13% in EV group (Table S2). 2-ethylhexanol, 2,3-butanediol and cis-4-decen-1-ol increased significantly by 60.97–103.39% in YB group, in contrast, isobutanol, 1-Butanol, isoamyl alcohol, 1-Pentanol, 3-Methyl-1-pentanol, 2-Ethylhexanol and phenethyl alcohol showed dramatically decrease of 21.42–44.69% in EV wine, only 1-nonanol significantly increased by 25.04%. Others alcohols, including 2-heptanol, 1-heptanol and 3-hexen-1-ol showed no significant differences among different wines (Figure 3D).

#### 3.5.4. Influence on Aldehydes, Ketones and Fatty Acids

7 aldehyde and ketone compounds were detected in 2023 vintages, while 2-heptanone was not found in YB treatment. Compared to the control wine, the contents of methyl heptenone, 2-undecanone and 2-octanone in YB wine significantly increased by 65.77%, 44.79% and 12.14%, respectively, while 2-undecanone significantly increased by 24.64% in the EV wine. Moreover, the YB wine showed a significant higher content of methylheptenone, decylaldehyde and 2-undecanone by 13.91–46.67% compared to the EV wine (Figure 3E).

10 fatty acids were detected among wine samples, the predominant fatty acids remained the same with 2022, while lauric acid was only identified in the CK wine. The contents of acetic acid and butyric acid in the YB wine significantly increased by 19.07% and 16.57%, respectively, whereas hexanoic acid, heptanoic acid and octanoic acid in EV group increased by 31.71–39.92%. Compared to YB group, EV group showed a significant decrease of 26.03% in acetic acid, accompanied by significant increases of 36.73–89.72% in hexanoic acid, heptanoic acid, octanoic acid and decanoic acid (Figure 3E).

#### 3.5.5. Influence on Terpenes, C_13_-Norisoprenoids, Hydrocarbons and Other Compounds

In 2023 wines, 11 compounds were identified, nerolidol and nerolic acid were only detected in the CK wines, while farnesol was not detected in YB treatment. In the YB group, linalool and geranyl acetone significantly increased by 11.58% and 161.04%, while isogeraniol and geraniol significantly decreased by 50.02% and 29.87% compared to the control wine. In the EV group, damascone and farnesol increased dramatically by 35.82% and 23.49%. Citronellol, nerol and *β*-ionone exhibited no significant differences among different wine samples (Figure 3F).

Seven hydrocarbons and five other compounds were identified. Naphthalene and phenol were only detected in the CK wine. Compared to the CK group, the YB group showed a significant decrease of 29.77% in dodecane, while hexadecane and 3-(methylthio)-1-propanol significantly increased by 84.24% and 24.27%, respectively. In the EV group, dodecane and 3-(methylthio)-1-propanol significantly decreased by 36.66% and 25.11% (Figure 3F).

### 3.6. Influence on Key Aroma Compounds and Aroma Wheel of Cabernet Gernischt Wine

The odor activity value (OAV), defined as the ratio of the concentration of an aroma compound to its olfactory threshold, is an important index for assessing overall aroma contribution. Orthogonal partial least squares-discriminant analysis (OPLS-DA) was performed to evaluate the aroma-active compounds with OAV > 0.1 in both vintages. A discriminant model was established based on the OAV values of key aroma components, and the score plot visually distinguished the aroma differences among wine samples from different treatments.

#### 3.6.1. Key Aroma Compounds of Cabernet Gernischt Wine in 2022 Vintage

**Figure 4:**
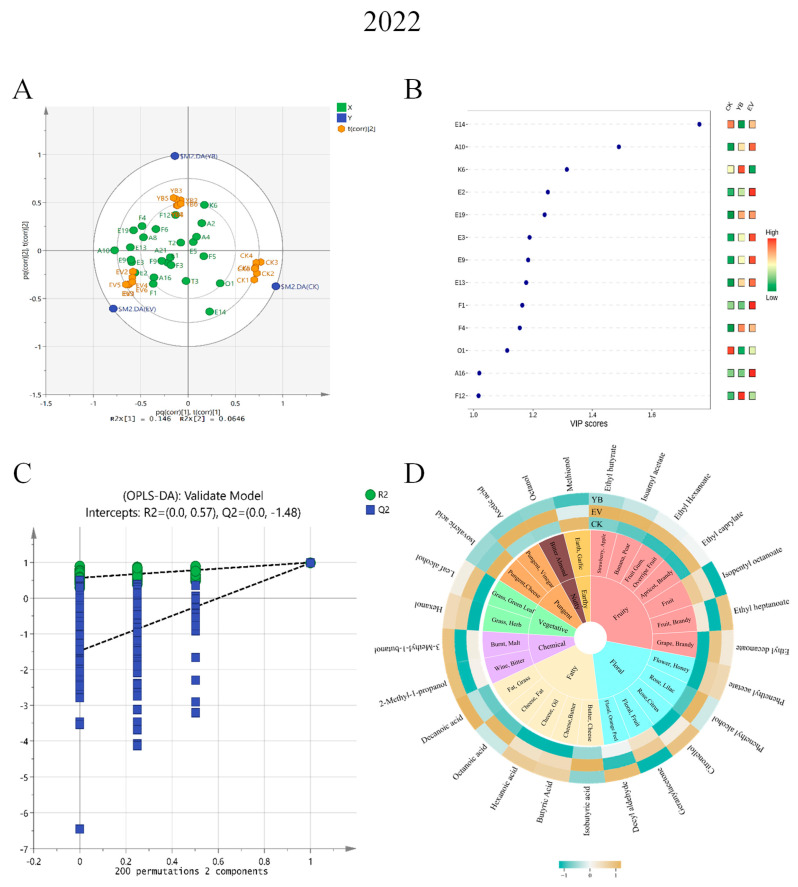
Impact on key aroma compounds and aroma wheel of Cabernet Gernischt wine in 2022 vintage. (**A**). Biplot of volatile compounds of OPLS-DA model, (**B**). Volatile compounds with VIP > 1, (**C**). Validate model of OPLS-DA analysis, (**D**). Aroma wheel of wine samples.

In 2022 vintage, the OPLS-DA score plot effectively separated CK wine from the YB and EV treatments. CK distributed in the fourth quadrant and closely correlated with isoamyl octanoate (E14) and 3-(methylthio)-1-propanol (O1). YB was located in the third quadrant and highly correlated with decylaldehyde (K6) and decanoic acid (F12). EV was also distributed in the fourth quadrant, and showed stronger correlations with 3-hexen-1-ol (A10), isoamyl acetate (E2), ethyl hexanoate (E3), ethyl octanoate (E9), ethyl decanoate (E13), acetic acid (F1) and 1-octanol (A16) (Figure 4A). A total 12 key aroma compounds were identified with VIP > 1, including isoamyl octanoate (E14), 3-hexen-1-ol (A10), decylaldehyde (K6), isoamyl acetate (E2) and phenethyl acetate (E19) (Figure 4B). Additionally, phenethyl acetate (E19), ethyl decanoate (E13) and butyric acid (F4) could also be regarded as marker substances to distinguish the control from treated wines, potentially providing stronger floral, fruity and fatty aroma notes in the COS wine samples.

After 200 permutation cross-validation, the R^2^X was 0.146, R^2^Y was 0.0646, the intercept of the Q_2_ regression line was -1.48, indicating that the model showed no overfitting, the validation was effective, and the analytical results were reliable (Figure 4C).

Compared with the control wine, the OAVs of 3-hexen-1-ol and phenethyl acetate in 2022 vintage significantly increased by 15.65–75.12% in both the YB and EV wines, with no significant differences observed between the two treatment groups. The OAV of isoamyl octanoate significantly increased by 57.65% in the EV wine samples, whereas it was not detected in the YB group. The OAVs of isoamyl acetate, ethyl hexanoate, ethyl octanoate, and ethyl decanoate significantly increased by 51.96–88.32% in the EV group, while butyric acid and decanoic acid significantly increased by 41.21–367.43% in the YB group. The OAV of decylaldehyde significantly increased by 60.27% in the YB group but significantly decreased by 39.92% in the EV group. Compared with the control group, the OAVs of acetic acid, 1-octanol, and methionol decreased by 2.80–13.95% or increased by 18.76–55.92%, although none of these changes reached statistical significance (Table S3).

#### 3.6.2. Aroma Wheel of Cabernet Gernischt Wine in 2022 Vintage

The aroma wheel could comprehensively characterize the overall aromatic style of wine by combining the odor activity value (OAV) and flavor characteristics of aroma compounds [26]. The results showed that Cabernet Gernischt wine presented multiple aroma attributes, including fruity, floral, chemical, pungent, microbial, fatty, vegetal and spicy notes (Figure 4D).

In 2022 vintage, fruity was the dominate aroma profile, mainly contributed by ethyl esters and acetate esters. The EV treatment exhibited higher OAVs of ethyl octanoate, ethyl hexanoate, isoamyl acetate, ethyl butyrate and ethyl decanoate, while the YB treatment presented higher OAVs of decylaldehyde and ethyl heptanoate. The control wine showed relatively lower OAVs of these key aroma compounds (Figure 4D).

Floral note was also an important aroma characteristic, mainly composed by terpenes, C_13_ norisoprenoids, and aromatic substances, presenting rose and clove notes. The EV treatment showed higher OAVs of phenethyl acetate, phenethyl alcohol and geranylacetone, while the YB group showed considerable OAVs of citronellol and phenethyl acetate, only geranyl acetone presented a relatively high OAV in the CK wine (Figure 4D).

Fatty notes ranked as the third dominant aroma attribute, mainly derived from fatty acids and octanol, exhibiting cheese and slight rancid characteristics. The YB group exhibited higher OAVs of butyric acid, hexanoic acid and decanoic acid, the EV group showed advantages in octanoic acid and octanol, while the CK wine maintained relatively lower levels (Figure 4D).

Vegetal notes were mainly contributed by C_6_ compounds. Interestingly, both COS treatment showed higher OAVs than the control wine. For chemical notes, the YB treatment presented with the highest OAVs of isobutanol and isoamyl alcohol, followed by the CK group, while the EV group with the lowest values. Pungent aroma mainly originated from acetic acid and isovaleric acid, which reached the highest OAVs in the EV group and the CK group, respectively. 3-(methylthio)-1-propanol, with earthy aroma, showed the highest value in the CK group (Figure 4D).

#### 3.6.3. Key Aroma Compounds of Cabernet Gernischt Wine in 2023 Vintage

In 2023 vintage, wine samples from different treatment also achieved satisfactory separation by OPLS-DA analisis.

**Figure 5:**
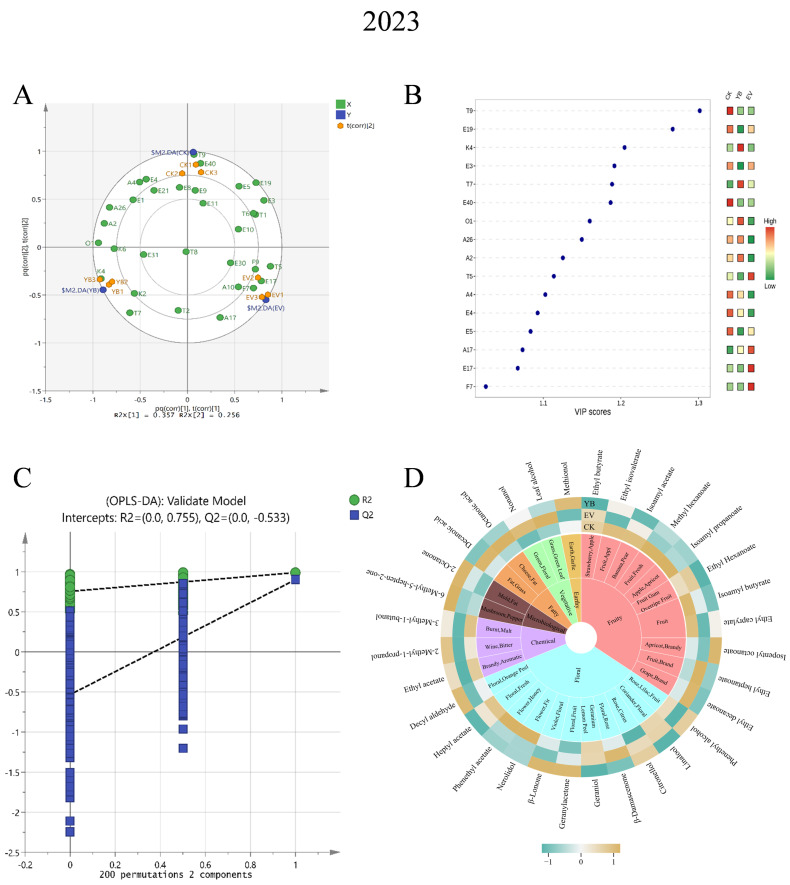
Impact on key aroma compounds and aroma wheel of Cabernet Gernischt wine in 2023 vintage. (**A**). Biplot of volatile compounds of OPLS-DA model, (**B**). Volatile compounds with VIP > 1, (**C**). Validate model of OPLS-DA analysis, (**D**). Aroma wheel of wine samples.

CK was distributed in the first and second quadrants and closely correlated with nerolidol (T9), phenethyl acetate (E40), isoamyl alcohol (A4), ethyl isovalerate (E4), isoamyl acetate (E5) and heptyl acetate (E19). YB was located in the third quadrant and exhibited high correlations with methylheptenone (K4), geranylacetone (T7), 3-(methylthio)-1-propanol (O1), decylaldehyde (K6) and 2-octanone (K2). EV was located in the fourth quadrant, and was strongly associated with β-damascenone (T5), 1-nonanol (A17), ethyl heptanoate (E17), octanoic acid (F7), decanoic acid (F9), 3-hexen-1-ol (A10) and ethyl decanoate (E30) (Figure 5A). A total of 16 key aroma compounds were indentified by VIP > 1, including nerolidol (T9), heptyl acetate (E19), methylheptenone (K4), ethyl butyrate (E3), geranylacetone (T7) and ethyl isovalerate (E4) (Figure 5B). Among these, heptyl acetate (E19), ethyl butyrate (E3) and isoamyl acetate (E5) could be used as key substances to distinguish wine samples treated at the YB stage, while phenethyl alcohol (A26) and isobutanol (A2) were considered differential components for the EV group (Figure 3B).

After 200 permutation cross-validation, the R^2^X was 0.357, R^2^Y was 0.256, the intercept of the Q_2_ regression line was −0.533, indicating that the model showed no overfitting, the validation was effective, and the analytical results were reliable (Figure 5C).

Among all the key aroma compounds, nerolidol was detected only in the control wine. Compared with the control wine, the OAVs of heptyl acetate and phenethyl acetate significantly decreased by 16.29–57.41% in both the YB and EV wines. The OAV of methionol significantly increased by 24.66% in the YB group but significantly decreased by 24.33% in the EV group. The OAVs of methyl heptenone and geranylacetone significantly increased by 56.11% and 195.36% in the YB group, while ethyl butyrate and isoamyl acetate significantly decreased by 68.98% and 39.60%, respectively. The OAVs of β-damascenone, 1-nonanol, ethyl heptanoate, and octanoic acid significantly increased by 23.23–31.44% in the EV group, whereas phenethyl alcohol, isobutanol, isoamyl alcohol, and ethyl isovalerate significantly decreased by 21.93–58.94% in the EV group (Table S3).

#### 3.6.4. Aroma Wheel of Cabernet Gernischt Wine in 2023 Vintage

The aroma wheel of 2023 vintage was similar to that of 2022, dominated by fruity and floral, but also presenting chemical, microbial, fatty and vegetal attributes. Fruity remained the primary dominant note, mainly contributed by ethyl esters and acetate esters. Compared to the CK group, the overall fruity aroma intensity decreased in COS-treated wines, among which the EV treatment showed higher OAVs of ethyl butyrate, ethyl hexanoate and heptyl acetate than the YB treatment (Figure 5D).

Floral also consisting of terpenes, C_13_ norisoprenoids, and aromatic compounds. The CK wine showed higher OAVs of nerolidol and phenethyl acetate, while the EV wine exhibited higher values of β-damascenone, nonanol and citronellol, the YB wine showed advantages in citronellol, *β*-ionone, geranylacetone and phenethyl alcohol (Figure 5D). Among other aroma attributes, the EV wine presented with lower OAVs of ethyl acetate, isobutanol and isoamyl alcohol related to chemical notes than CK and YB wines, while possessing higher OAVs of decanoic acid and 3-hexen-1-ol associated with fatty and vegetal notes. The YB wine showed obvious advantages in OAVs of microbial characteristic substances including 2-octanone and methylheptenone (Figure 5D).

These results indicated that, pre-harvest chitooligosaccharide treatment significantly altered the aroma notes of wine, and exhibited obvious vintage effect. Comprehensive comparison indicated that early veraison treatment presented better effect on wine aroma quality.

## 4. Discussion

### 4.1. Chitooligosaccharide Treatment Influenced Physico-Chemical Components and Color Parameters of Cabernet Gernischt Wine

Pre-harvest chitooligosaccharide treatment exhibited diverse effects on the weight, the °Brix and total acidity of grape berries, as well as the alcohol content, total acidity and phenolic profiles of Cabernet Gernischt wine in 2022 and 2023 vintages. These effects can be attributed to the indirect modulation of gene expression and metabolic pathways in grapes, which further modifies physicochemical parameters of berries and the resulting wine. Another possible explanation is that elicitors application may led to a delay in grape maturity [27], particularly in early veraison treatment of 2023 vintage. Consistently, Qiang et al. [18] demonstrated that chitooligosaccharide could activate the phenylpropanoid pathway in grape berries, thereby stimulating the biosynthesis of phenolic compounds. Our previous study observed that the content of total phenols, tannins and anthocyanins was higher in chitooligosaccharide treated grapes skins, however, this superior quality did not transmitted to the final wine in 2023 vintage, which is presumed to be closely associated with the altered cell wall structure of grape skins [28].

Similar research indicated that pre-harvest BTH treatment increased skin cell wall thickness by 1.63- to 3.79-fold in Cabernet Gernischt grapes, with the most remarkable effect observed at the berry expansion stage [21]. Other studies also confirmed that exogenous application of plant growth regulators during fruit development could upregulate the activities of cell wall-metabolizing enzymes and the expression of related genes, promoted the accumulation of lignin and hydroxyproline-rich glycoproteins, and ultimately increase the thickness and mechanical rigidity of grape skin cell walls [28,29]. These structural changes inevitably restrain the release and extraction of skin derived phenolic compounds during alcoholic fermentation. Therefore, appropriate optimization of maceration conditions is recommended to improve the extraction efficiency of phenolic substances from grape skins in winemaking.

Notably, the changes in anthocyanin content showed significantly positive correlations with color saturation (C*) and red-green chromaticity coordinate (a*) of the wine. Liu [17] reported that foliar application of yeast extract resulted in significantly higher L* and b* values, but lower a* and C*ab values in Cabernet Sauvignon wines. Similar trends were observed in this study, compared to the control wines, chitooligosaccharide treated wines exhibited significantly higher L*and b*, while the a* and C*decreased, suggesting that the color of wines became lighter with a more pronounced yellow hue. Although such effects varied with application stages and vintages, the variations in all of the above color parameters were positively correlated with anthocyanin content in wine samples, further confirmed that the regulatory effect of chitooligosaccharide on wine color is primarily mediated by anthocyanin extraction and accumulation.

However, during the fermentation process, in order to minimize the influence from micro-vinification, all grapes of each treatment were fermented together without biologically repetition, this tends to a limitation of this study. In future, pilot-scale fermentation with biologically repetition should be induced.

### 4.2. Chitooligosaccharide Application Exhibit Varied Effects on Volatile Composition and Aroma Substances of Cabernet Gernischt Wine

Pre-harvest treatment with plant growth regulator affects the composition and volatile substances by regulating fatty acids, amino acids and isoprene metabolic pathways in grape berries, and has been proved as an effective technical to modulate and improve wine aroma and sensory quality [16,22,23].

Terpenoids and C_13_ norisoprenoids are the main contributors to the floral and fruity aromas of wine, although their content in red grape variety is relatively low. The application of salicylic acid (SA) significantly promoted the free and bound monoterpenes accumulation in mature Muscat Hamburg grapes by increasing the transcription of some key genes related to monoterpene biosynthesis. Consequently, the concentrations of geraniol, neral, geranic acid, nerol, nerol oxide, and phellandrene were higher in wines produced from grapes treated with 50 mg/L SA than the control groups. Onofrio et al. confirmed that pre-harvest methyl jasmonate (MeJA) treatment significantly increased the total concentration of several berry aroma classes by approximately two-fold, from about 3 to 6 μg/g in berries. Moreover, the total content of free monoterpenoids in Sangiovese wine produced by microvinification increased by 7.39 times, with an significant impact on wine longevity and sensorial characters [27]. Liu et al. [17] reported that foliar sprays of yeast extract (YE) increased total norisoprenoids by 28% in Cabernet Sauvignon berries and by 82% in Marselan berries. However, volatiles in Cabernet Sauvignon wines lost 29–42% of ethyl isobutyrate, ethyl 2-methylbutyrate, ethyl acetate, and geranyl acetone, flattening fruity and floral perceptions, whereas the same esters increased 46–55% in Marselan wines, intensifying ripe black-fruit notes [17]. These findings indicated a cultivar-specific strategy for tailoring red-wine style and aroma structure.

The above research results are consistent with the conclusions of this study. In 2022 vintage, the total content of esters in chitooligosaccharide treated wines significantly increased by 34.72–53.60%, among which the terpenoid content in the YB group significantly increased by 161.04%. In 2023 vintage, the fatty acid content in the EV group significantly increased by 32.70%, although no significant difference was observed in the total terpenoid content, significant differences were found in the contents of key terpenoid components such as linalool and geranylacetone, indicating a vintage effect on concentration on the concentration of most volatile compounds in wines. Gutiérrez-Gamboa also demonstracted that the concentration of terpenoids and C_13_ norisoprenoids in Tempranillo blanco wine was more affected by season rather than by seaweed applications [30].

In the present study, an aroma wheel was used to evaluate the influence of pre-harvest chitooligosaccharide spraying on wine aroma quality. However, the threshold values of some aroma compounds were not available, leading to a certain discrepancy between the constructed aroma wheel and the actual sensory profile of the wine samples. Further studies are required to clarify the aromatic characteristics and dose-effect relationships of key aroma substances [25]. In addition, gas chromatography-olfactometry (GC-O) should be used to identify key aroma profile of Cabernet Gernischt wines, and recombination and omission test of key aroma notes should also be performed.

### 4.3. Pretreatment Methods Produced Varied Effects on Volatile Components and Aroma Quality

The concentration of volatile substances is one of the key issues in the qualitative and quantitative analysis of wine aroma compounds [3]. Dispersive liquid-liquid microextraction (DLLME) showed the advantages of simple operation, low cost and high concentration factor, while headspace solid-phase microextraction (HS-SPME) is advantageous due to its convenient operation, high extraction efficiency and environmental friendliness [24,31]. The influence of these methodological differences on wine aroma analysis results warrants in-depth discussion.

In this study, DLLME and HS-SPME were used to analyze the volatile compounds in Cabernet Gernischt wines from 2022 and 2023 vintages. The results showed that both methods effectively detected the aroma substances but exhibited significant differences. HS-SPME identified a greater number of volatile components and performed better in detecting esters and terpenoids, which may be related to its extraction selectivity for low-boiling and highly volatile compounds. In contrast, DLLME showed greater advantages for high-boiling components such as higher alcohols, fatty acids and hydrocarbons, accompanied by higher quantitative precision. This superiority is presumably attributed to its mechanism, which is more suitable for the separation and extraction of substances with high polarity and high boiling points [31]. Accordingly, multiple pretreatment methods should be applied to compensate for the limitations of any single method, thereby enabling a more comprehensively characterization of variations in aroma components in future studies.

## 5. Conclusions

This study demonstrates that pre-harvest chitooligosaccharide (COS) spraying significantly affects the physicochemical parameters, phenolic compounds, color index, sensory profiles, volatile composition, key aroma compounds, and aroma wheel attributes of Cabernet Gernischt wine.

In 2022 vintage, alcohol content and total acidity increased in both treatment groups. Total phenols increased by 11.94% in the EV group but decreased by 14.67% in the YB group. Lightness and yellow tone increased, whereas red tone and color saturation decreased. No significant differences were observed in sensory evaluation. The total volatile content increased by 8.18%, with terpenoids increasing by 138.91% (YB group) and total esters by 34.72–53.60% in COS-treated wines. Phenethyl acetate, ethyl caprate, and butyric acid were identified as key aroma compounds distinguishing COS-treated wines from the control. Fruity and floral notes were enhanced, with the EV group showing superior characteristics.

In 2023 vintage, the YB and EV treatments exhibited opposite effects on alcohol content and total acidity. Total phenols, tannins, and anthocyanins significantly decreased by 17.65–35.25%. Color index showed similar trends to those observed in 2022. No significant differences were found in sensory evaluation among the treated wines. Total volatile content decreased by 15.36–15.76%, with esters dropping by 26.60% and alcohols by 25.96% in the YB group, while fatty acids increased by 32.70% in the EV group. Heptyl acetate, ethyl butyrate, and isoamyl acetate were identified as key aroma compounds distinguishing the YB group, whereas phenethyl alcohol and 2-methyl-1-propanol were typical for the EV group. Fruity and floral notes were reduced, although the EV group showed less decreases.

Although vintage-dependent variations exist, chitooligosaccharide application at the early veraison stage represents a simple, economical, and controllable viticultural strategy for improving Cabernet Gernischt wine quality. Future studies should optimize application dosage to maximize the benefits in practical production.

## Figures and Tables

**Table 1:** Oenological parameters of Cabernet Gernischt wine.

Vintages	2022			2023		
Treatment	CK	YB	EV	CK	YB	EV
Weight of 100 berries (g)	146.84 ± 1.97 a	148.77 ± 2.84 a	168.18 ± 0.71 b	194.70 ± 2.95 b	181.97 ± 4.61 a	186.60 ± 1.85 a
°Brix	20.13 ± 0.06 a	20.93 ± 0.32 b	21.57 ± 0.15 c	21.20 ± 0.36 a	24.80 ± 0.35 b	21.17 ± 0.76 a
Total acidity(g/kg)	6.23 ± 0.15 ns	5.93 ± 0.31 ns	5.97 ± 0.15 ns	7.63 ± 0.50 b	6.60 ± 0.10 a	7.03 ± 0.06 a
Alcohol (%)	11.18 ± 0.02 a	11.21 ± 0.01 a	11.59 ± 0.01 b	11.59 ± 0.01 b	12.33 ± 0.01 c	10.53 ± 0.01 a
Total acidity (g/L)	8.41 ± 0.02 a	8.55 ± 0.01 b	8.78 ± 0.01 c	7.24 ± 0.03 b	6.65 ± 0.03 a	7.55 ± 0.02 c
pH	3.59 ± 0.01 ns	3.57 ± 0.00 ns	3.57 ± 0.00 ns	3.49 ± 0.00 a	3.83 ± 0.01 b	3.43 ± 0.00 a
Residual sugar(g/L)	0.22 ± 0.11 a	0.50 ± 0.11 b	0.42 ± 0.04 b	0.38 ± 0.1 b	0.1 ± 0.05 a	0.05 ± 0.06 a
Volatile acidity (g/L)	0.21 ± 0.04 a	0.22 ± 0.01 a	0.24 ± 0.01 b	0.29 ± 0.1 a	0.45 ± 0.05 b	0.58 ± 0.06 c
Total phenols (mg/L)	795.33 ± 13.23 b	678.67 ± 21.28 a	890.33 ± 4.41 c	1102.00 ± 10.14 b	1098.11 ± 10.84 b	906.44 ± 8.55 a
Total tannins (mg/L)	451.82 ± 19.16 b	286.36 ± 3.15 a	454.85 ± 15.89 b	609.39 ± 8.97 b	614.24 ± 12.11 b	459.09 ± 6.30 a
Total anthocyanins (mg/L)	169.29 ± 13.51 ns	162.57 ± 4.61 ns	176.01 ± 7.99 ns	230.59 ± 4.06 c	189.89 ± 3.47 b	149.30 ± 2.13 a

Treatments: CK—Control Check, YB—Young Berry stage, EV—Early Veraison stage. Total acidity is expressed as the content of tartaric acid. Different letters within columns in each vintage indicate significant differences between the treatments, ns means no significant difference were observed (Tukey’s range test, *p* < 0.05).

**Table 2:** CIE Lab color parameters of Cabernet Gernischt wine.

Vintage	2022			2023		
Treatment	CK	YB	EV	CK	YB	EV
Lightness (L*)	89.40 ± 0.21 a	92.79 ± 0.29 c	90.11 ± 0.13 b	85.65 ± 0.33 a	87.74 ± 0.02 b	91.56 ± 0.22 c
Green-red axis (a*)	10.17 ± 0.17 b	7.32 ± 0.26 a	10.36 ± 0.04 b	19.54 ± 0.11 c	15.39 ± 0.19 b	11.23 ± 0.27 a
Blue-yellow axis (b*)	−0.39 ± 0.07 a	0.29 ± 0.05 b	0.28 ± 0.07 b	0.50 ± 0.14 a	1.03 ± 0.12 b	0.48 ± 0.08 a
Chromaticity (C*ab)	10.18 ± 0.16 b	7.33 ± 0.27 a	10.37 ± 0.04 b	19.55 ± 0.11 c	15.42 ± 0.18 b	11.24 ± 0.27 a
Hue (H*ab)	−0.038 ± 0.007 a	0.040 ± 0.006 c	0.027 ± 0.007 b	0.026 ± 0.007 a	0.067 ± 0.008 c	0.043 ± 0.006 b

Different letters within columns in each vintage indicate significant differences between the treatments (Tukey’s range test, *p* < 0.05).

## Data Availability

The original contributions presented in this study are included in the article/Supplementary Material. Further inquiries can be directed to the corresponding authors.

## Supplementary Materials

The following supporting information can be downloaded at: https://www.mdpi.com/article/10.3390/foods15122128/s1, Figure S1: Correlation analysis of phenolic content and CIELab parameters in wine samples; Figure S2: Average Temperature in 2022 and 2023 vintage; Figure S3. Average Rainfall in 2022 and 2023 vintage; Table S1. Volatile compounds of Cabernet Gernischt Wine in 2022 vintage; Table S2. Volatile compounds of Cabernet Gernischt Wine in 2023 vintage; Table S3. Key aroma compounds and OAV value in Cabernet Gernischt wine.

## Abbreviations

The following abbreviations are used in this manuscript:

OPLS-DA	Orthogonal least squares-discriminant analysis
OAV	Odor activity value
DLLME	Dispersive liquid-liquid microextraction
HS-SPME	headspace solid-phase microextraction
GC-MS	Gas chromatography-mass spectrometer
COS	Chitooligosaccharide
