# Supplementary material for: Pre-Harvest Foliar Application of Chitooligosaccharide Modulates Aroma Quality of Cabernet Gernischt Wines
Source: Foods. 2026 Jun 12;15(12):2128. doi: 10.3390/foods15122128 (PMC13297753; doi:10.3390/foods15122128)
Supplement: Supplementary file 1 [file foods-15-02128-s001.zip › foods-4342381-supplementary figures.pdf]

Supplementary figures

Figure 4D. Aroma wheel of Cabernet Gernischt wine in 2022 vintage

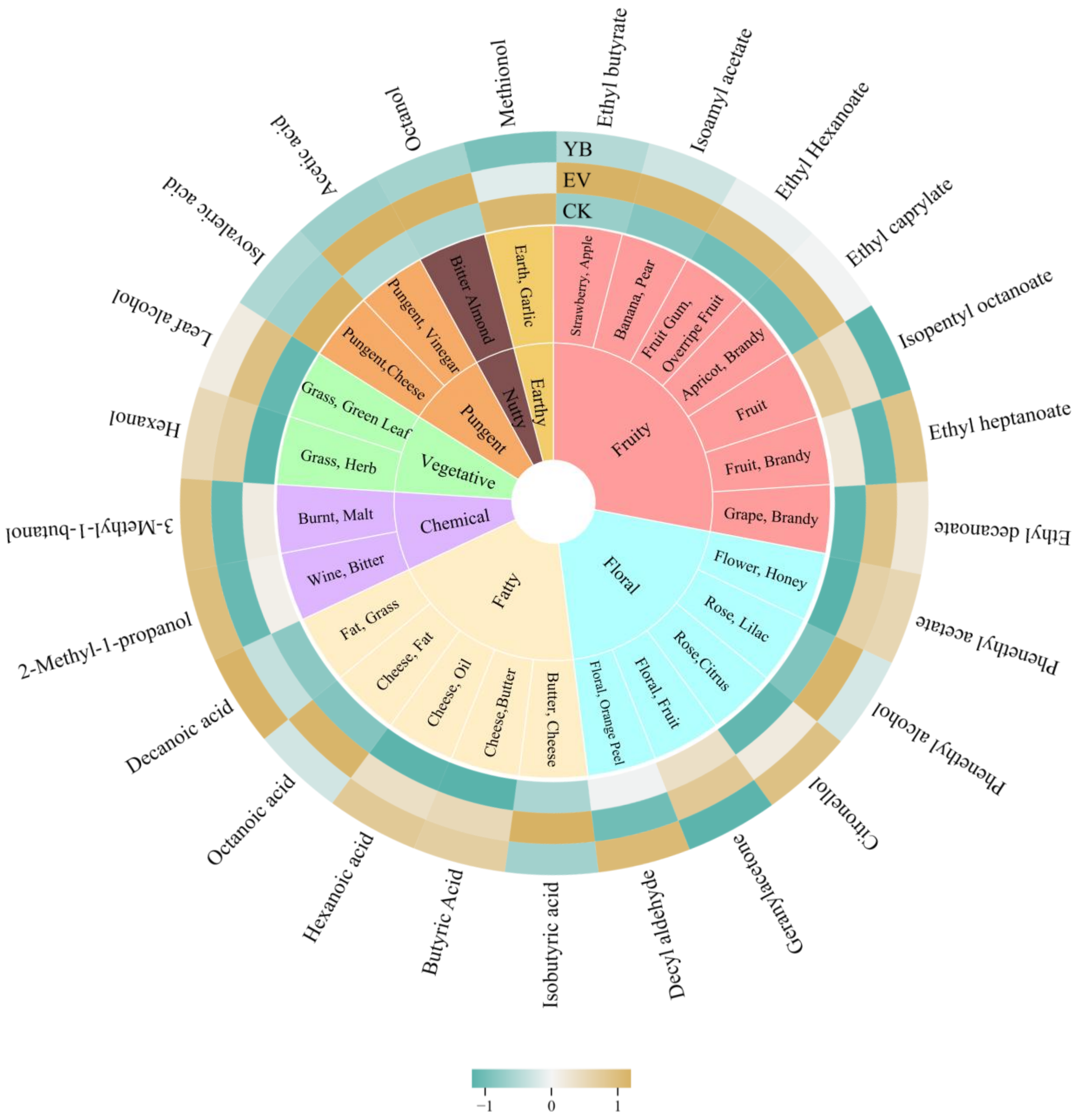

Figure 4D. Aroma wheel of Cabernet Gernischt wine in 2022 vintage

Figure 5D. Aroma wheel of Cabernet Gernischt wine in 2023 vintage

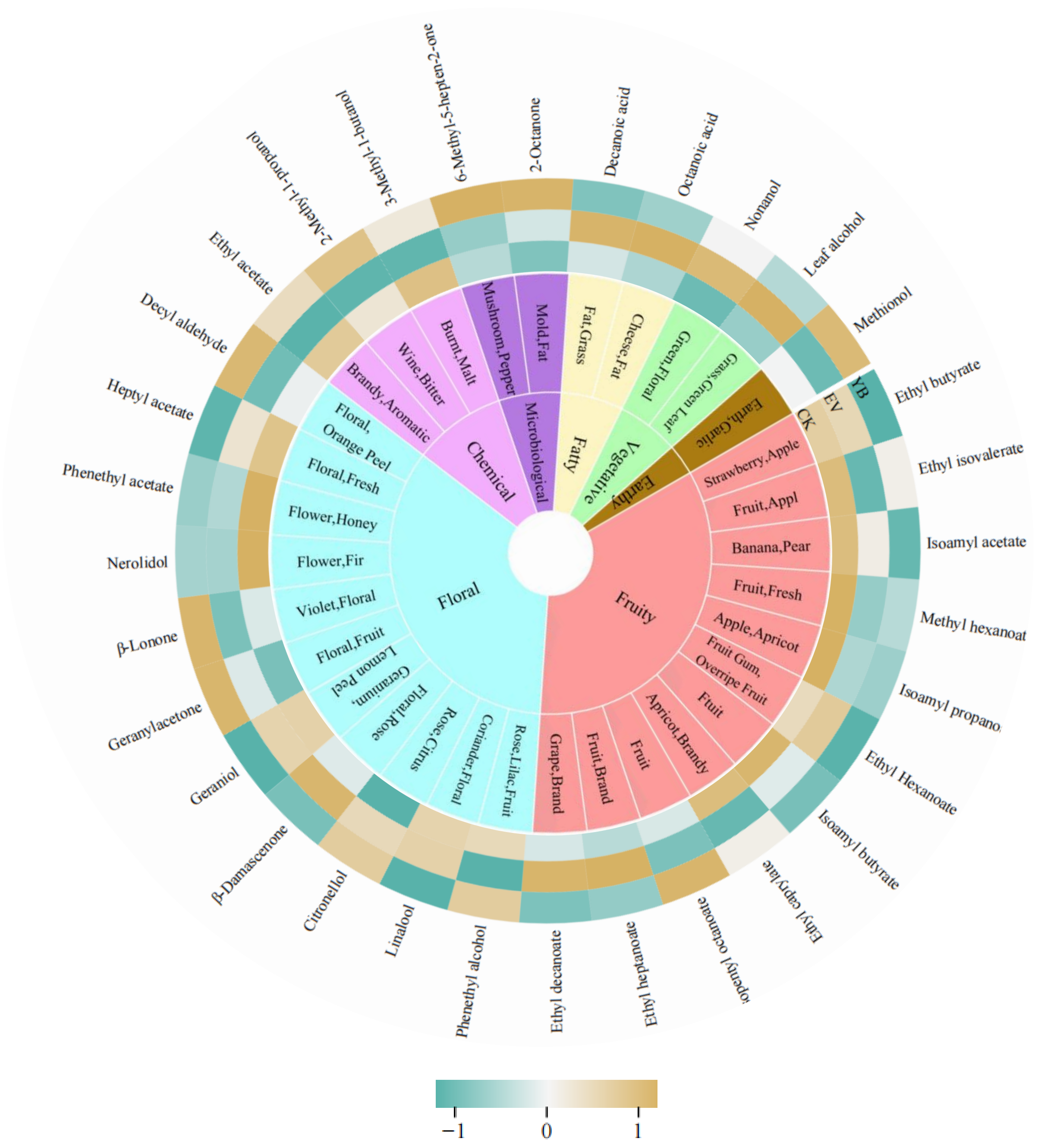

Figure 5D. Aroma wheel of Cabernet Gernischt wine in 2023 vintage

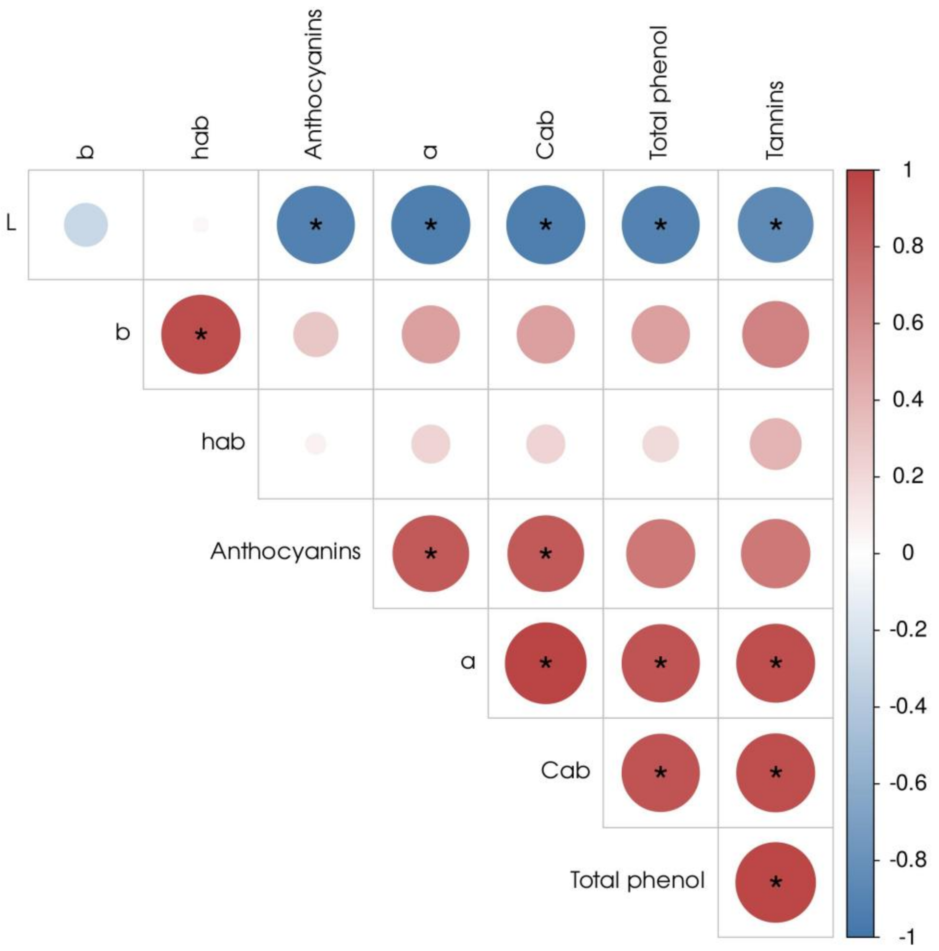

Fig. S1. Correlation analysis of phenolic content and CIELab parameters in wine samples

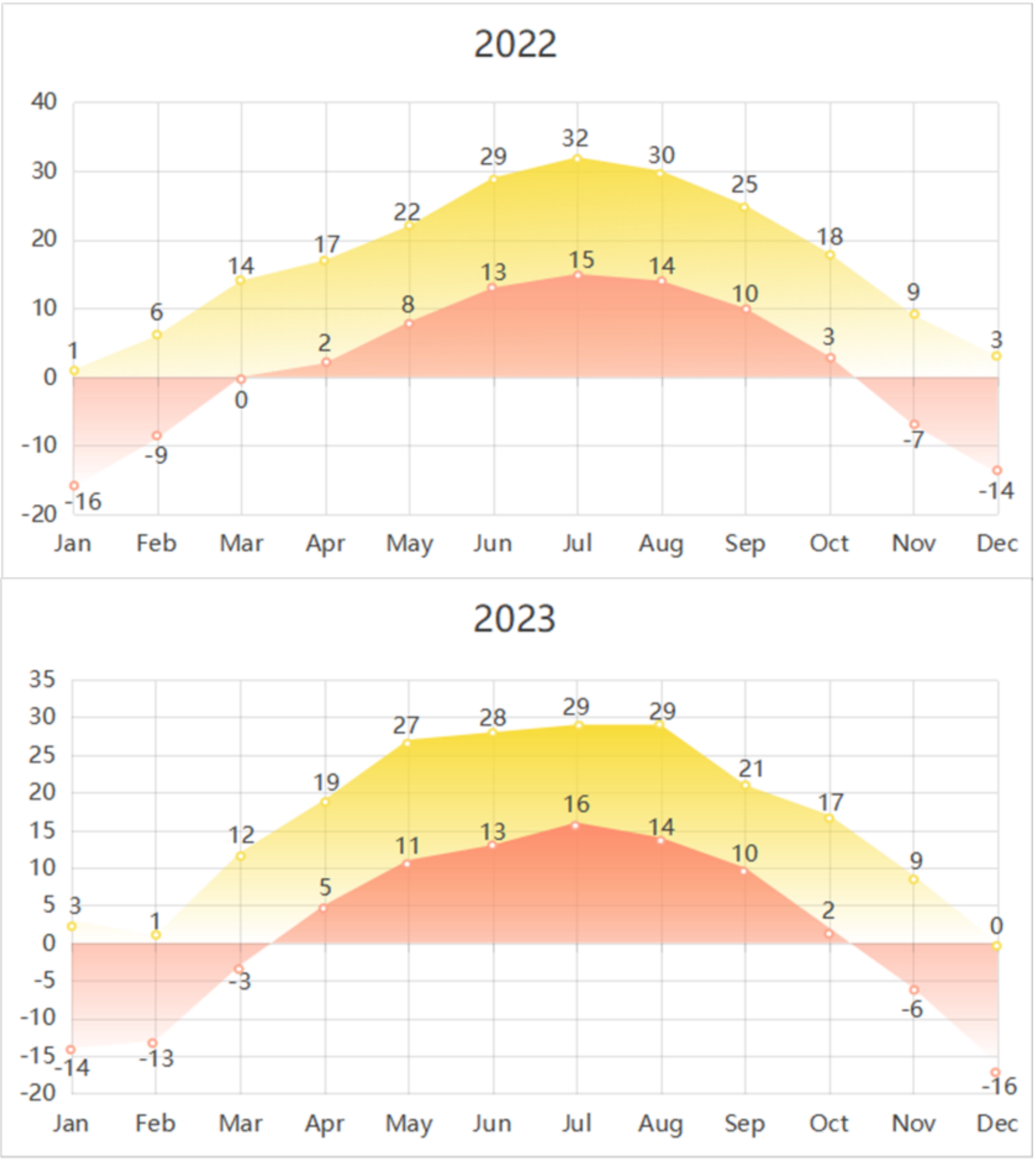

Fig. S2. Average Temperature in 2022 and 2023 vintage

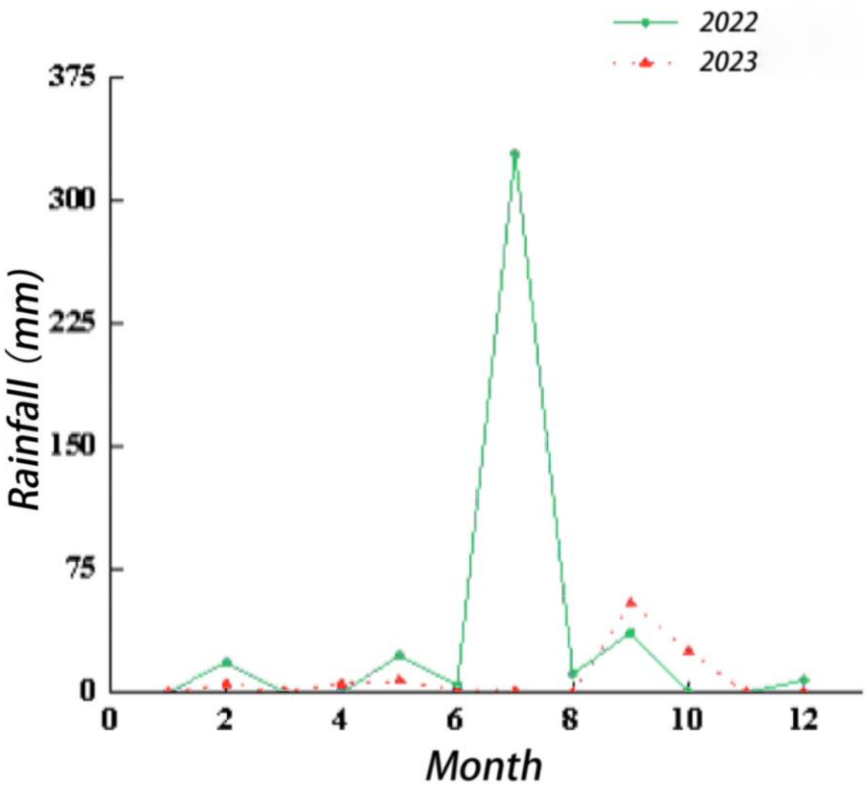

Fig. S3. Average Rainfall in 2022 and 2023 vintage
