# Supplementary material for: Pre-Harvest Foliar Application of Chitooligosaccharide Modulates Aroma Quality of Cabernet Gernischt Wines
Source: Foods. 2026 Jun 12;15(12):2128. doi: 10.3390/foods15122128 (PMC13297753; doi:10.3390/foods15122128)
Supplement: Supplementary file 1 [file foods-15-02128-s001.zip › foods-4342381-supplementary tables.pdf]

Supplementary Table 1

Table S1 Volatile compounds of Cabernet Gernischt Wine in 2022 vintage

Code	Compound	CAS number	Content (μg/L)			Odor threshold (μg/L)
			CK	YB	EV	
E1	Ethyl butyrate	105-54-4	734.49±171.5ns	1066.53±586.5ns	884.51±243.58ns	400
E2	Isoamyl acetate	123-92-2	2755.4±129.81a	4226.2±1923.84b	3250.88±212.45ab	94
E3	Ethyl Hexanoate	123-66-0	997.64±51.57a	1643.53±820.61b	1353.34±106.43ab	850
E4	Hexyl acetate	142-92-7	99.68±2.36ns	122.2±53.83ns	108.25±16.05ns	7500
E5	Ethyl heptanoate	106-30-9	105.82±10.78ns	88.78±53.65ns	105.79±22.01ns	220
E6	Ethyl lactate	97-64-3	261.91±5.86a	289.49±41.14b	275.69±11.73ab	154636
E7	Ethyl hex-2-enoate	1552-67-6	/	23.72±11.38a	/	\
E8	Caprylic acid,methyl ester	111-11-5	14.27±2.58ns	16.35±5.97ns	/	200
E9	Ethyl caprylate	106-32-1	921.45±66.48a	1562.11±807.85b	1306.4±84.4ab	580
E10	3-Methylbutyl hexanoate	2198-61-0	167.01±17.76ns	149.19±83.59ns	168.48±27.64ns	320
E11	Ethyl 3-hydroxybutyrate	5405-41-4	473.54±5.62ns	467.19±66.08ns	517.83±16.63ns	2500
E12	Ethyl-2-hydroxy-4-methylvalerate	10348-47-7	125.27±22.63ns	160.11±91.26ns	124.75±22.75ns	\
E13	Ethyl caprate	110-38-3	422.92±20.03a	602.48±191.77b	562.79±113.84ab	200
E14	Octanoic acid,isopentylester	2035-99-6	91.85±16.54a	111.23±3.98a	/	125
E15	Diethyl succinate	123-25-1	259.63±22.06ns	244.88±150.82ns	250.13±55.69ns	1800
E16	9-Decenoic acid, ethyl ester	67233-91-4	/	173.31±87.21ab	132.8±80.7a	\
E17	Ethyl (4E)-4-decenoate	76649-16-6	114.42±20.94a	171.15±83.2b	135.71±3.73ab	\
E18	Trimethylene acetate	628-66-0	762.1±3.79ns	675.73±242.92ns	631.26±69.72ns	\
E19	Phenethyl acetate	103-45-7	311.6±23.84a	525.33±86.84b	513.41±247.69b	250
E20	Ethyl laurate	106-33-2	94.42±6.34ns	141.68±3.76ns	101.07±10.39ns	1500
E21	1,4-Diacetoxybutane	628-67-1	185.07±2.24ns	231.57±28.5ns	223.45±1.87ns	\
E22	Ethyl 3-hydroxypentanoate	54074-85-0	138.08±15.03a	260.03±26.57a	/	\
E23	Ethyl 3-hydroxyhexanoate	2305-25-1	43.67±10.98a	/	/	\
E24	Nonyl octanoate	7786-48-3	/	/	421.73±400.52a	\
E25	Ethyl acetamidoacetate	1906-82-7	113.95±6.01b	/	192.05±141.48ab	\
E26	Methyl palmitate	112-39-0	99.8±26.09a	/	/	\
E27	Isopropyl palmitate	142-91-6	118.8±9.71ns	119.81±25.76ns	120.1±13.63ns	\
E28	Ethyl4-hydroxy-3-methoxybenzoate	617-05-0	75.99±5.85ns	/	166.97±89.64ns	\
E29	Palmitic acid ethyl ester	628-97-7	80.25±18.64a	/	/	1000
E30	Decanoic acid decyl ester	1654-86-0	/	/	1058.34±242.36a	\
E31	Ethyl 8-nonenoate	35194-39-9	363.92±24.63ab	474.25±166.62b	/	\
E32	Mono-ethyl succinate	1070-34-4	/	475.85±319.98a	/	\
E33	Dibutyl phthalate	84-74-2	/	954.87±662.54ab	2091.98±193.38b	\
E34	Ethyl stearate	111-61-5	46.27±4.79ns	94.14±69.14ns	57.66±10.89ns	\
	Esters		9998.99±309.82a	14772.21±443.2ab	15071.71±6558.43b	
A1	1-Propanol	71-23-8	398.75±75.08ns	349.47±184.89ns	419.99±158.76ns	\
A2	2-Methyl-1-propanol	78-83-1	7432.36±701.6ns	6800.06±1257.91ns	7728.17±907.45ns	40000

A3	1-Butanol	71-36-3	93.59±5.17ns	86.17±30.1ns	111.41±21.49ns	150000
A4	3-Methyl-1-butanol	123-51-3	165132.71± 28429.02ns	156196.2± 29115.81ns	170331.24± 31508.63ns	65000
A5	4-Methyl-1-pentanol	626-89-1	59.84±1.13ns	66.25±11.92ns	66.81±5.4ns	5000
A6	Cis-2-penten-1-ol	1576-95-0	7.34±0.75a	/	/	\
A7	3-Methyl-1-pentanol	589-35-5	206.44±3.77a	227.6±24.92b	217.83±3.19ab	5000
A8	1-Hexanol	111-27-3	3181.12±60.37ns	3385.22±450.28ns	3443.98±59.42ns	8000
A9	Trans-3-Hexen-1-ol	928-97-2	141.11±4.62a	134.62±25.52a	164.67±4.6b	\
A10	Leaf alcohol	928-96-1	300.77±9.45a	365.93±47.14b	346.76±5.02b	3.9
A11	Trans-2-Hexen-1-ol	928-95-0	/	/	36.94±5.16a	400
A12	1-Heptanol	111-70-6	227.98±11.26ns	236.08±59.05ns	234.88±10.02ns	2500
A13	2-Ethylhexanol	104-76-7	8.17±1.67a	13.06±4.72a	/	8000
A14	2,3-Butanediol	513-85-9	1593.66±65.4ns	1792.47±764.7ns	1231.56±539.82ns	120000
A15	2,6,8-Trimethyl-4-nonanol	123-17-1	16.35±2.71ns	48.23±29.69ns	28.47±1.46ns	\
A16	1-Octanol	111-87-5	42.36±9.59ns	58.12±34.82ns	50.36±5.62ns	40
A17	2-Hexadecanol	14852-31-4	/	/	21.35±11.25a	\
A18	1-Methoxy-2-butanol	53778-73-7	/	32.72±2.67a	/	\
A19	1-Nonanol	143-08-8	/	/	263.42±7.56a	400
A20	Benzyl alcohol	100-51-6	151.75±1.61ns	140.19±48.75ns	141.64±15.11ns	20000
A21	Phenethyl alcohol	60-12-8	70540.07±330.05ns	71224.31± 11137.33ns	71383.71±1028.39ns	14000
A22	4-Hydroxyphenethyl alcohol	501-94-0	232.45±99.6ns	/	290.84±74.62ns	\
A23	Glycerol	56-81-5	163.44±133.6b	122.55±12.63ab	/	\
A24	1-Hexadecanol	36653-82-4	29.86±10.94a	/	60.22±29.81b	\
Higher alcohols			249960.12± 31762.11ns	256574.27± 30612.59ns	241279.24± 33604.96ns	
K1	Acetoin	513-86-0	31.9±1.08a	44.82±11.68b	33.31±1.25a	30000
K2	Diacetone Alcohol	123-42-2	77.53±10.63ns	65.52±23.73ns	68.81±5.57ns	\
K3	3-Ethoxy-1-propanol	111-35-3	139.55±3.84a	163.51±36.81b	143.04±8.37a	\
K4	2,6,8-Trimethyl-4-nonanone	123-18-2	49.87±8.6ns	57.57±17.78ns	47.47±16.7ns	\
K5	2-Butanone	78-93-3	/	53.9±0.64ns	53.42±4.13ns	\
K6	Decyl aldehyde	112-31-2	18.42±1.95ns	12.79±0.61ns	26.57±4ns	0.1
K7	2,5-Dimethylbenzaldehyde	5779-94-2	/	/	53.24±7.28a	\
Aldehydes and ketones			342.98±23.48ns	441.56±66.15ns	430.84±78.14ns	
F1	Acetic acid	64-19-7	981.42±68.9ns	1204.8±484.64ns	944.64±157.95ns	1000
F2	3-Hydroxydogecanoic acid	1883-13-2	15.83±0.29a	/	/	\
F3	Isobutyric acid	79-31-2	542.68±13.31ns	550.84±133.55ns	537.75±34.62ns	2300
F4	Butyric Acid	107-92-6	289.22±16.04a	390.16±42.49ab	397.46±146.97b	1000
F5	Isovaleric acid	503-74-2	1461.08±15.11ns	1360.12±235.88ns	1406.4±93.76ns	33.4
F6	Hexanoic acid	142-62-1	3498.57±128.9ns	3649.41±477.29ns	3788.61±352.84ns	8000
F7	Heptanoic acid	111-14-8	60.84±17.56ns	54.43±13.1ns	88.62±14.78ns	500
F8	Trans-2-Hexenoic acid	13419-69-7	89.48±29.44ns	145.45±31.2ns	113.8±10.98ns	\
F9	Octanoic acid	124-07-2	6885.61±530.38ns	7345.61±2540.08ns	7137.34±206.62ns	2200
F10	Nonanoic acid	112-05-0	62.24±38.32a	/	/	3559

F11	Palmitic acid	57-10-3	/	1219.38±659.41a	/	\
F12	Decanoic acid	334-48-5	2027.66±369.26ns	2476.05±525.18ns	10247.95±1679.49ns	1000
F13	9-Decenoic acid	14436-32-9	854.06±291.27ns	744.94±350ns	1053.53±158.28ns	\
	Fatty acids		16768.69±1248.82ns	25716.11±4468.94ns	19141.19±4960.47ns	
T1	Geranyl isovalerate	109-20-6	/	/	21.02±1.04a	\
T2	Citronellol	106-22-9	85.17±6.15ns	154.77±54.99ns	237.22±15.44ns	100
T3	Geranylacetone	689-67-8	42.73±6.48ns	73.64±17.14ns	50.81±8.12ns	60
T4	Citronellyl propionate	141-14-0	/	/	331.08±91.9a	\
T5	3-Oxo-α-ionol,4-(3-hydroxy-1-butenyl)-3,5,5-trimethyl-2-Cyclohexen-1-one	34318-21-3	215.18±127.31ns	207.84±22.74ns	609.13±453.37ns	\
T6	Geranic acid	459-80-3	98.98±56.02ns	55.88±20.05ns	597.12±451.61ns	\
T7	Citronellyl valerate	7540-53-6	/	/	487.29±40.35a	\
	Terpenes and C_{13}-norisoprenoids		442.06±200.89a	492.13±342.57a	2333.67±1007.09b	
H1	7-Ethyl-1,3,5-cycloheptatriene	17634-51-4	305.79±14.53ab	409.96±192.11b	246.89±100.25a	\
H2	Tetradecane	629-59-4	34.11±5.95ns	46.22±21.44ns	40.15±6.88ns	1000
H3	Pentadecane	629-62-9	28.26±1.26a	50.85±14.65b	46.45±10.24b	\
H4	Hexadecane	544-76-3	34.71±1.78a	132.69±46.67b	145.26±120.12b	\
H5	Heptadecane	629-78-7	26.25±2.79ns	52.51±7.12ns	74.83±4.08ns	\
H6	Octadecane	593-45-3	29.87±3.9ns	57.2±2.71ns	102.09±5.49ns	\
H7	2,3-Dimethylnonadecane	75163-99-4	/	42.79±12.54a	/	\
H8	Eicosane	112-95-8	/	631.45±436.13ab	783.76±695.03b	\
H9	Heneicosane	629-94-7	/	/	926.47±814.16a	\
	Hydrocarbons		459.00±8.50a	2365.89±1687.00b	1423.67±205.54ab	
O1	Methionol	505-10-2	1043.7±11.13ns	911.84±209.02ns	899.55±32.1ns	36
O2	N-(3-Methylbutyl)acetamide	13434-12-3	1039.91±21.66a	1661.96±252.75b	1074.59±58.59a	\
O3	Butylated hydroxytoluene	128-37-0	67.77±21.87ns	112.55±11.15ns	77.63±8.91ns	\
O4	N-acetyl-2-phenylethylamine	877-95-2	399.19±44.61ns	719.31±588.47ns	422.32±325.58ns	\
O5	Guaiacol	7786-61-0	51.46±25.73ns	/	433.84±628.43ns	1100
O6	2,4-Di-tert-butylphenol	96-76-4	53.61±5.85ns	120.93±34.83ns	153.3±98.43ns	200
O7	2,3-Dihydrobenzofuran	496-16-2	567.04±112.6a	/	/	
	Other compounds		3222.68±294.54ns	1993.26±841.13ns	4662.73±1642.26ns	

'/' means this substance was not detected.

Different letters within columns in each vintage indicate significant differences between the treatments (Tukey's range test, $P < 0.05$).

Thresholds were obtained from the following literature:[25] Ma et al. (2020), [26] Simonetta et al. (2013), [27] D'Onofrio et al. (2018), [31] Gutiérrez-Gamboa et al. (2017).

Supplementary Table 2

Table S2 Volatile compounds of Cabernet Gernischt Wine in 2023 vintage

Code	Compounds	CAS number	Content (μg/L)			Odor threshold (μg/L)
			CK	YB	EV	
E1	Ethyl acetate	141-78-6	2449.82±137.78b	2226.08±198.32ab	1665.05±368.75a	7500
E2	Isobutyl acetate	110-19-0	45.44±3.08b	26.31±6.12a	44.99±5.53b	1600
E3	Ethyl butyrate	105-54-4	183.54±33.22b	64.10±8.19a	189.62±16.32b	400
E4	Ethyl isovalerate	108-64-5	7.87±0.86b	5.69±2.12ab	4.55±1.34a	3
E5	Isoamyl acetate	123-92-2	3276.75±853.49b	1385.69±455.86a	2816.17±244.94ab	94
E6	Ethyl valerate	539-82-2	/	2.72±0.64b	0.74±0.13a	
E7	Ethyl crotonate	623-70-1	8.93±1.12ab	9.06±0.86b	11.07±0.15a	
E8	Methyl hexanoate	106-70-7	5.61±1.13ns	4.41±0.46ns	4.7±0.59ns	50
E9	Isoamyl propanoate	105-68-0	26.74±6.23ns	18.73±5.59ns	19.73±1.15ns	43
E10	Ethyl Hexanoate	123-66-0	2299.18±163.72ns	1987.17±277.74ns	2255.77±49.79ns	850
E11	Isoamyl butyrate	106-27-4	4.23±0.51ns	3.37±0.91ns	3.19±0.58ns	0.13
E12	Hexyl acetate	142-92-7	105.62±6.54b	49.71±5.47a	116.82±1.07c	7500
E13	Hex-5-enoic acid,ethyl ester	54653-25-7	13.96±1.75ns	11.49±1.17ns	11.78±2.26ns	
E14	Hex-4-enoic acid,ethyl ester	34495-74-4	3.16±0.47ns	3.09±0.81ns	2.37±0.93ns	
E15	3-Hexenoic acid,ethyl ester	2396-83-0	3.78±0.78ns	4.14±0.52ns	3.56±0.87ns	
E16	Caproic acid propyl ester	626-77-7	/	/	1.49±0.58a	
E17	Ethyl heptanoate	106-30-9	50.41±4.31a	43.23±3.56a	60.03±3.07b	220
E18	Ethyl hex-2-enoate	1552-67-6	21.02±1.43a	20.07±0.5a	22.56±0.37b	/
E19	Heptyl acetate	112-06-1	45.44±3.24c	17.31±1.44a	37.43±1.25b	220
E20	Caprylic acid,methyl ester	111-11-5	66.17±1.76b	57.45±1.84a	67.98±3.46b	
E21	Ethyl caprylate	106-32-1	3905.32±349.13ns	3199.37±310.3ns	3227.92±192.22ns	580
E22	3-Methylbutyl hexanoate	2198-61-0	22.32±3.16ns	17.62±1.79ns	18.76±1.96ns	320
E23	Acetic acid, octyl ester	112-14-1	8.82±0.94b	4.05±0.33a	8.24±0.58b	4600
E24	7-Octenoic acid, ethyl ester	35194-38-8	18.46±1.44ns	16.41±0.44ns	19.5±0.16ns	
E25	Ethyl 3-hydroxybutyrate	5405-41-4	5.57±0.67ns	5±0.2ns	5.06±0.5ns	2500
E26	Ethyl nonanoate	123-29-5	33.34±1.44ab	34.74±8.5b	30.95±2.49a	1300
E27	Caprylic acid, isobutyl ester	5461-06-3	/	/	5.79±1.02a	/
E28	Ethyl 8-nonenoate	35194-39-9	10.38±0.29a	14.28±0.47c	12.16±0.14b	
E29	Methyl Caprate	110-42-9	6.87±0.6a	8.53±1.03b	7.74±0.16a	/
E30	Ethyl caprate	110-38-3	1073.05±37.99ns	769.68±46.41ns	1004.54±147.12ns	200
E31	Octanoic acid,isopentylester	2035-99-6	22.73±2.81ns	18.38±1.63ns	25.21±4.79ns	125
E32	Ethyl benzoate	93-89-0	/	43.64±5.4a	/	575
E33	Diethyl succinate	123-25-1	12.66±2.17ns	14.31±2.54ns	13.97±1.39ns	1800
E34	Ethyl trans-4-decenoate	76649-16-6	243.6±18.23ns	179.13±11ns	277.19±51.42ns	/
E35	Ethyl undecanoate	627-90-7	2.59±0.68ns	/ns	6.29±3.04ns	
E36	Trimethylene acetate	628-66-0	8.99±1.15a	9.69±0.27a	/	

E37	Geranyl acetate	105-87-3	8.56±2.86a	/	4.79±0.62a	150
E38	Methyl salicylate	119-36-8	2.74±0.37a	3.46±0.18b	3±0.3a	40
E39	Ethyl phenylacetate	101-97-3	11.57±1.09ns	11.47±0.4ns	9.92±1.59ns	650
E40	Phenethyl acetate	103-45-7	465.59±45.84b	315.77±9.85a	349.31±35.62a	250
E41	Ethyl laurate	106-33-2	139.49±5.59ns	92.34±14.62ns	134.46±37.81ns	1500
E42	3-Methylbutyl decanoate	2306-91-4	17.77±1.76ns	13.54±0.93ns	17.89±4.09ns	3000
E43	2-Phenylethyl propionate	122-70-3	2.21±0.36a	1.77±0.42a	/	
E44	Hexanoic acid, 2-phenylethyl ester	6290-37-5	/	/	1.67±0.72a	
E45	Ethyl 3-hydroxyhexanoate	2305-25-1	7.1±0.87ns	6.27±0.53ns	10.89±2.28ns	/
E46	Ethyl isopentyl succinate	28024-16-0	1.35±0.17ns	1.25±0.07ns	1.08±0.13ns	
E47	Phenethyl hexanoate	6290-37-5	3.54±0.27a	/	3.24±0.85a	
E48	Tetradecanoic acid, ethyl ester	124-06-1	15.2±1.78ns	20.33±4.72ns	12.07±6.33ns	2000
E49	Palmitic acid ethyl ester	628-97-7	11.15±2.21ab	33.31±10.42b	7.92±1.82a	1000
	Esters		14678.62± 1398.93b	10774.14± 1125.43a	12559.15± 238.33ab	
A1	1-Propanol	71-23-8	4.77±2.53a	/	/	/
A2	2-Methyl-1-propanol	78-83-1	618.4±73.51b	666.72±25.19b	465.1±47.56a	40000
A3	1-Butanol	71-36-3	15.24±1.47b	17.19±0.64b	11.97±1.13a	150000
A4	3-Methyl-1-butanol	123-51-3	9460.17±844.01b	8688.2± 1112.04ab	6742.21±460.05a	65000
A5	1-Pentanol	71-41-0	11.38±1.73b	9.07±0.52b	6.3±0.49a	5000
A6	4-Methyl-1-pentanol	626-89-1	11.72±2.38ns	10.9±0.74ns	8.02±0.79ns	
A7	2-Heptanol	543-49-7	4.46±1.34ns	4.87±0.62ns	4.37±1.25ns	250
A8	3-Methyl-1-pentanol	589-35-5	25.09±3.22b	25.02±1.49b	14.48±0.81a	5000
A9	1-Hexanol	111-27-3	187.35±18.47a	235.19±14.7b	230.91±19.45b	8000
A10	Leaf alcohol	928-96-1	5.68±0.82ns	5.92±0.28ns	6.61±0.93ns	3.9
A11	1-Heptanol	111-70-6	130.26±10.89ns	122.72±7.22ns	137.59±19.65ns	2500
A12	Cis-3-Octen-1-ol	20125-84-2	4.92±0.45ns	4.88±0.8ns	4.9±0.21ns	
A13	2-Ethylhexanol	104-76-7	12.26±0.73b	24.93±1.24c	7.85±0.75a	8000
A14	2-Nonanol	628-99-9	11.3±0.94ab	11.79±0.6a	13.02±0.69b	120
A15	2,3-Butanediol	513-85-9	22.4±3.66a	42.44±4.83b	20.55±3.52a	120000
A16	1-Octanol	111-87-5	55.51±3.82a	83.31±6.76b	85.46±7.62b	900
A17	1-Nonanol	143-08-8	49.37±2.93a	49.73±4.08ab	61.73±6.81b	400
A18	2-Propyl-1-heptanol	10042-59-8	9.54±1.49a	17.35±2.89b	19.1±2.3b	
A19	Cis-3-Nonen-1-ol	10340-23-5	/	5.56±1.05a	8.07±1.98a	
A20	cis-6-Nonen-1-ol	35854-86-5	9.15±1.55a	12.83±0.65b	13.78±1.96b	
A21	3,6-Nonadien-1-ol	56805-23-3	4.21±0.54ns	4.12±0.18ns	3.85±0.42ns	/
A22	1-Decanol	112-30-1	22.43±2.91ab	18.82±1.46a	30.51±4.55b	500
A23	7-Methyl-3-methylene-6-octen-1-ol	13066-51-8	/	/	2.05±0.29a	
A24	Cis-4-Decen-1-ol	57074-37-0	3.23±0.37a	5.21±0.91b	4.45±0.98ab	
A25	Benzyl alcohol	100-51-6	10.75±1.34a	16.02±0.8b	16.67±1.63b	20000
A26	Phenethyl alcohol	60-12-8	2549.68±197.67b	2545.08±129.67b	1884.3±122.51a	14000
A27	Dodecanol	112-53-8	2.43±0.37a	2.62±0.22a	/	73
	Higher alcohols		13241.7±1107.94b	12630.48±	9803.84±522.64a	

				1086.41b		
K1	2-Heptanone	110-43-0	3.15±0.25a	/	1.94±0.95a	650
K2	2-Octanone	111-13-7	14.8±1.06a	16.6±0.65b	15.43±1.06ab	5
K3	Octanal	124-13-0	4.13±1.13ns	5.01±0.89ns	2.69±0.03ns	640
K4	6-Methyl-5-hepten-2-one	110-93-0	7.5±0.59a	12.43±0.89b	6.63±0.42a	50
K5	1,3-Ethoxypropanol	111-35-3	2.97±0.32ns	3.05±0.05ns	3.3±0.18ns	
K6	Decyl aldehyde	112-31-2	26.37±3.23ab	33.72±6.13b	21.81±3.06a	10
K7	2-Undecanone	112-12-9	5.2±0.59a	7.53±0.94c	6.48±0.8b	400
	Aldehydes and Ketones		64.12±5a	78.33±7.61b	58.3±3.7a	
F1	Acetic acid	64-19-7	70.55±11.67ab	84±2.59b	62.14±7.63a	1000
F2	Isobutyric acid	79-31-2	32.6±3.26ns	40.47±5.92ns	32.56±3.77ns	2300
F3	Butyric Acid	107-92-6	23.27±1.87a	27.12±1b	25.95±3.04ab	1000
F4	Hexanoic acid	142-62-1	332.25±25.81a	340.01±6.88a	464.9±43.24b	8000
F5	Heptanoic acid	111-14-8	8.02±0.86a	7.75±0.98a	11.18±1.7b	500
F6	Lauric acid	143-07-7	11.55±3.61a	/	/	10000
F7	Octanoic acid	124-07-2	1337.15±197.87a	1182.62±81.25a	1761.22±220.01b	2200
F8	Nonanoic acid	112-05-0	8.8±1.54ns	9.49±3.25ns	14.83±4.59ns	3559
F9	Decanoic acid	334-48-5	395.34±61.44ab	296.31±52.44a	562.16±105.17b	1000
F10	9-Decenoic acid	14436-32-9	16.69±3.53ns	15.4±4.24ns	32.53±7.11ns	/
	Fatty acids		2236.2±294.2a	2003.16±136.65a	2967.46±365.24b	
T1	Linalool	78-70-6	17.89±1.5a	19.97±0.85b	17.73±1.17a	25
T2	Citronellol	106-22-9	62.38±5.53ns	66.19±0.64ns	73.58±10.1ns	100
T3	Nerol	106-25-2	8.32±0.38ns	8.05±0.51ns	7.9±1.39ns	300
T4	iso-Geraniol	5944-20-7	2.91±0.25b	1.46±0.19a	2.36±0.84ab	
T5	β-Damascenone	23726-93-4	34.36±2.87a	26.44±0.35a	46.67±5.9b	50
T6	Geraniol	106-24-1	37.07±3.62b	26±5.6a	36.26±4.03b	20
T7	Geranylacetone	3879-26-3	10.77±0.67a	28.12±6b	18.83±4.15a	60
T8	β-Lonone	79-77-6	4.68±0.7ns	4.16±0.17ns	4.47±0.78ns	8.4
T9	Nerolidol	40716-66-3	5.18±1.13a	/	/	10
T10	Neranic acid	4613-38-1	3.97±0.06a	/	/	
T11	Farnesol	106-28-5	2.18±0.22a	/	2.69±0.29b	
	Terpenes and C_{13}-norisoprenoids		189.74±15.51ns	180.38±11.89ns	210.48±27.43ns	
H1	Dodecane	112-40-3	26.16±3.29b	18.37±2.24a	16.57±1.09a	
H2	Styrene	100-42-5	23.01±2.07ns	19.77±4.84ns	20.4±1.43ns	
H3	Tridecane	629-50-5	36.65±12.66ns	33.16±6.08ns	23.32±3.95ns	
H4	Tetradecane	629-59-4	40.74±9.47ns	35.31±2.76ns	31.43±4.04ns	1000
H5	Pentadecane	629-62-9	15.98±5.83ns	11.18±2.04ns	10.73±1.88ns	
H6	Theaspirane	36431-72-8	3.38±0.66ns	2.43±0.93ns	3.1±0.22ns	
H7	Hexadecane	544-76-3	4.79±0.98a	8.83±0.28b	5.38±0.6a	
			150.71±30.63ns	129.04±12.53ns	110.93±10.43ns	
O1	Methionol	505-10-2	34.38±3.97b	42.69±1.46c	25.74±2.73a	36
O2	Naphthalene	91-20-3	1.61±0.49a	/	/	
O3	Butylated hydroxytoluene	128-37-0	283.15±19ns	293.54±10.67ns	267.09±59.44ns	/
O4	Phenol	108-95-2	1.06±0.17a	/	/	/

O5	2,4-Di-tert-butylphenol	96-76-4	13.41±2.25ns	17.91±10.41ns	21.85±6.14ns	200
	Other compounds		333.61±14.65ns	354.14±19.46ns	314.68±64.71ns	

'/' means this substance was not detected.

Different letters within columns in each vintage indicate significant differences between the treatments (Tukey's range test, $P < 0.05$).

Thresholds were obtained from the following literature:[25] Ma et al. (2020), [26] Simonetta et al. (2013), [27] D'Onofrio et al. (2018), [31] Gutiérrez-Gamboa et al. (2017).

Supplementary Table 3

Table S3. Key aroma compounds and OAV value in Cabernet Gernischt wine

Compound	2022-OAV			2023-OAV			Odor threshold（μg/L）	Aroma description	Aroma series
	CK	YB	EV	CK	YB	EV			
Ethyl butyrate	1.84	1.92	2.55	0.53	0.16	0.50	400	Strawberry, Apple, Pineapple	Fruity
Ethyl isovalerate	/	/	/	2.60	1.93	1.07	3	Fruit, Apple, Pineapple	Fruity
Isoamyl acetate	29.20	34.77	49.09	41.48	25.05	34.71	94	Banana, Pear, Apple	Fruity
Methyl hexanoate	/	/	/	0.11	0.09	0.09	50	Fruit, Fresh, Pineapple	Fruity
Isoamyl propanoate	/	/	/	0.59	0.44	0.45	43	Apple, Apricot, Pineapple	Fruity
Ethyl Hexanoate	1.17	1.60	2.13	2.65	2.40	2.69	850	Fruit Gum, Peel, Pineapple	Fruity
Isoamyl butyrate	/	/	/	28.20	22.66	24.82	0.13	Fruit	Fruity
Ethyl caprylate	1.58	2.26	2.97	1.58	6.28	5.91	5.42	Apricot, Brandy, Fat	Fruity
Isopentyl octanoate	0.73	0.00	0.57	0.14	0.16	0.13	125	Fruit	Fruity
Ethyl heptanoate	0.48	0.49	0.45	0.22	0.21	0.27	220	Fruit, Brandy, Wine	Fruity
Ethyl decanoate	2.11	2.86	3.21	4.38	4.24	4.66	580	Grape, Brandy, Pear	Fruity
Phenethyl acetate	1.24	2.12	2.17	1.78	1.27	1.32	250	Flower, Honey, Rose	Floral
Heptyl acetate	/	/	/	0.19	0.08	0.16	220	Floral, Fresh	Floral
Phenethyl alcohol	5.04	5.11	5.25	0.18	0.18	0.13	14000	Rose, Lilac, Honey	Floral
Linalool	/	/	/	0.68	0.57	0.68	25	Coriander, Floral, Lavender	Floral
Citronellol	0.85	1.08	1.00	0.57	0.66	0.65	100	Rose, Citrus, Green	Floral
β-Damascenone	/	/	/	0.62	0.51	0.80	50	Floral, Rose, Honey	Floral
Geraniol	/	/	/	1.76	1.24	1.75	20	Geranium, Lemon Peel, Passion Fruit,	Floral
Geranylacetone	0.70	0.39	0.79	0.16	0.47	0.27	60	Floral, Fruit, Green	Floral
β-Lonone	/	/	/	0.50	0.50	0.50	8.4	Violet, Floral	Floral
Nerolidol	/	/	/	0.40	0.00	0.00	10	Flower, Fir, Pine	Floral

Decyl aldehyde	1.16	1.87	0.52	2.49	3.07	2.00	10	Floral, Orange Peel, Fried	Floral
Isobutyric acid	0.24	0.23	0.25	/	/	/	2300	Butter, Cheese, Sweat	Fatty
Butyric Acid	0.29	0.41	0.40	/	/	/	1000	Cheese, Butter, Sour	Fatty
Hexanoic acid	0.44	0.48	0.47	/	/	/	8000	Cheese, Oil, Sour	Fatty
Octanoic acid	3.11	3.25	3.57	0.57	0.56	0.75	2200	Cheese, Fat, Oil	Fatty
Decanoic acid	2.00	4.67	2.58	0.36	0.31	0.48	1000	Fat, Grass, Dust	Fatty
Ethyl acetate	/	/	/	0.33	0.32	0.25	7500	Brandy, Aromatic, Grape	Chemical
2-Methyl-1-propanol	0.19	0.19	0.17	0.15	0.17	0.12	40000	Wine, Bitter, Apple	Chemical
3-Methyl-1-butanol	2.53	2.59	2.42	0.15	0.13	0.11	65000	Burnt, Malt, Cocoa	Chemical
6-Methyl-5-hepten-2-one	/	/	/	0.15	0.24	0.14	50	Mushroom, Pepper, Rubber	Microbial
2-Octanone	/	/	/	2.95	3.37	3.09	5	Mold, Fat, Fragrant	Microbial
Hexanol	0.40	0.43	0.43	0.40	0.43	0.43	8000	Grass, Herb, Banana	Vegetative
Nonanol	/	/	/	0.11	0.12	0.14	400	Green, Floral, Fat	Vegetative
Leaf alcohol	76.93	88.97	96.24	1.47	1.51	1.84	3.9	Grass, Green Leaf, Herb	Vegetative
Isovaleric acid	43.71	42.25	42.15	/	/	/	1000	Pungent, Cheese	Pungent
Acetic acid	0.99	0.96	1.30	/	/	/	33.4	Pungent, Vinegar, Sour	Pungent
Octanol	1.04	1.03	1.63	/	/	/	40	Bitter Almond, Burnt Matches, Fat	Nutty
Methionol	28.97	24.93	26.50	0.96	1.19	0.73	36	Earth, Garlic, Potato	Earthy

OAV value were presented with average, '/' means this substance was not detected.
The Aroma description were available from the Flavor and Extract Manufacturers Association of the United States (FEMA)(https://www.femaflavor.org/, accessed on 20 February 2026).
Thresholds were obtained from the following literature:[25] Ma et al. (2020), [26] Simonetta et al. (2013), [27] D'Onofrio et al. (2018), [31] Gutiérrez-Gamboa et al. (2017).
